# Sleep and cognitive outcomes in multiple sclerosis; a systematic review

**DOI:** 10.1186/s12888-024-06103-5

**Published:** 2024-09-28

**Authors:** Behnam Golabi, Hadis Razmaray, Sepideh Seyedi-Sahebari, Heliya Bandehagh, Zahra Hakimzadeh, Ailin Khosroshahi, Seyedehyasmin Moghaddamziabari, Negar Aghaei, Sarvin Sanaie, Mahnaz Talebi, Amirreza Naseri

**Affiliations:** 1 Student Research Committee, Tabriz University of Medical Sciences, Golgasht Street, Tabriz, East Azerbaijan, 5166/15731 Iran; 2 Neurosciences Research Center (NSRC), Tabriz University of Medical Sciences, Tabriz, 5166/15731 Iran; 3 Davis School of Medicine, University of California, Davis, Davis, CA United States of America; 4 Faculty of Medicine, Tabriz Medical Sciences, Islamic Azad University, Tabriz, Iran; 5 Research Center for Evidence-Based Medicine, Iranian EBM Centre: A Joanna Briggs Institute (JBI) Center of Excellence, Tabriz University of Medical Sciences, Tabriz, Iran; 6 Tabriz USERN Office, Universal Scientific Education and Research Network (USERN), Tabriz, Iran

**Keywords:** Cognitive dysfunction, Cognition, Sleep, Multiple sclerosis, Systematic review

## Abstract

**Background:**

Multiple sclerosis (MS) is a disabling disease of the central nervous system. People living with MS often have co-existing sleep disorders and cognitive dysfunction. The objective of this study was to scrutinize the relationship between cognitive outcomes and sleep conditions in MS.

**Methods:**

This study followed the Joanna Briggs Institute’s (JBI) and PRISMA guidelines. PubMed, Scopus, Embase, and Web of Science databases were searched and original studies delineating the relationship between sleep status and cognitive findings in MS patients‌ were included. The risk of bias was assessed using the JBI critical appraisal tools.

**Results:**

In the final review, out of 1635 screened records, 35 studies with 5321 participants were included. Pittsburgh Sleep Quality Index (PSQI), Epworth Sleepiness Scale (ESS), and polysomnography were the most common assessment tools for evaluation of sleep condition, and cognitive evaluations were conducted using the tests including Paced Auditory Serial Addition Test (PASAT), California Verbal Learning Test (CVLT), Symbol Digit Modalities Test (SDMT) and Brief Visuospatial Memory Test (BVMT). Assessing the quality of studies showed no significant bias in most of the included articles. A link between sleep condition and cognitive abilities was suggested in the literature, especially with objective measurement of sleep condition; however, current evidence did not support a substantial association between self-reported sleep quality and processing speed and working memory in patients with MS.

**Discussion:**

Evidence proposes sleep is an independent factor associated with cognitive outcomes in MS. Given the limitations of the evidence such as the lack of well-designed prospective studies, these findings need to be interpreted with caution.

## Introduction

 Multiple sclerosis (MS) is a disabling condition of the central nervous system (CNS). Signs and symptoms and severity of nerve fiber damage in the CNS vary widely between patients. Fatigue, sensory abnormalities, visual abnormalities, spasticity, pain, depression and anxiety, bladder problems, cerebellar dysfunction, and cognitive dysfunction are the most common manifestations of MS [1]. The incidence of MS is increasing worldwide, in line with the socioeconomic impact of the disease [2]. Relapsing-remitting (RRMS) which is characterized by relapses with stable neurological disability between episodes, primary progressive (PPMS) with a progressive course at onset, and secondary progressive (SPMS) which is a progressive course following an initial relapsing-remitting course, are the phenotypes of MS [3, 4]. As a result of advancements in comprehending the pathogenesis and course of this condition, pharmacological and non-pharmacological strategies for the management of MS symptoms [5–8], as well as effectual disease-modifying therapies [9–11], and supplementations [12, 13] are widely researched and found to be influential in enhancing patients’ quality of life and survival [14, 15].

Sleep disturbance is a general term for a wide spectrum of sleep-related symptoms and disorders, including insomnia, restless legs syndrome, sleep-disordered breathing, narcolepsy, and rapid eye movement (REM) sleep behavior disorder [16]. Low sleep quality and insufficient sleep during adolescence are suggested to increase the risk of developing MS [17]. Sleep disturbance is a prevalent manifestation in MS patients, even in patients with a low level of disability [18]. Poor sleep quality [19, 20], restless legs syndrome, and insomnia [21] are found to be more prevalent in MS [22]. A recent systematic review of polysomnographic findings revealed a considerable reduction in stage N2 sleep and sleep efficiency as well as increases in wake time after sleep onset, the periodic limb movement index, and the periodic limb movement arousal index in patients with MS [23]. Sleep abnormalities are reported to be significantly associated with other debilitating symptoms of the disease, such as fatigue, and can harm substantially patients’ quality of life [24].

On the other side, the literature on cognitive outcomes in MS patients has grown exponentially over the last few years, so that cognitive dysfunction is now recognized as a core symptom of MS [25]. Slowed information process speed, and episodic memory decline as well as impaired verbal fluency, learning abilities, executive function, and visuospatial memory are common among MS patients [26–28]. A recent systematic review reported 32.5% prevalence of cognitive dysfunction among patients with RRMS [29]. As one of several behavioral states, sleep can affect cognition. Sleep can provide conditions and make unique contributions to memory formation [30], which is not possible in arousal states. Through this, a possible connection between sleep status and cognitive function is suggested in the literature [31, 32]. In addition, daytime sleepiness is also found to correlate with cognitive status [33, 34].

A systematic review was conducted in 2018 to assess the relationship between sleep disturbance and cognitive dysfunction in MS patients [35], and found a significant correlation between sleep disorders and cognitive impairment. Researchers included 12 studies that were published until the final search in June 2017 all revealed significant associations between sleep disturbance and cognitive dysfunction. Due to growing interest in this contest in recent years, several studies were conducted to assess this correlation on different occasions, therefore, the present systematic review is designed to dissect the possible association between any sleep-related measurement and cognitive status, in patients with MS.

## Methods

This systematic review is conducted following the methods mentioned in the Joanna Briggs Institute’s (JBI) Manual for Evidence Synthesis [36], and the Preferred Reporting Items for Systematic Reviews and Meta-Analyses (PRISMA) statement [37].

### Eligibility criteria

All original clinical studies delineating the relationship between sleep status and cognitive findings in MS patients were included in this systematic review. Review articles, editorials, commentaries, conference abstracts, animal studies, in vitro studies, and non-English publications were excluded.

### Information sources and search strategy

Studies were identified through a systematic search of Scopus, Embase, PubMed, and Web of Science databases and carried out by two independent reviewers (A.N., S.M.). The last search was run on July 27, 2023 and updated via handsearching in August 2024. The following strategy was utilized for search in PubMed database: (“sleep“[MeSH Terms] OR “sleep“[All Fields] OR “sleeping“[All Fields] OR “sleeps“[All Fields] OR “sleep s“[All Fields]) AND (“Cognitive Dysfunction“[MeSH Terms] OR “cogni*“[All Fields]) AND (“Multiple Sclerosis“[MeSH Terms] OR “Multiple Sclerosis“[All Fields]). In addition, the references and citations of included studies and the related review articles as well as PubMed related articles were manually checked.

### Selection process

Retrieved studies were imported into Endnote and de-duplicated. Moreover, screening was fulfilled independently by two reviewers in title/abstract (H.R., S.S-S., S.M.) and full-text stages (A.N., B.G.) using the Rayyan Intelligent Systematic Review tool [38]. Discrepancies were resolved by discussion or decided upon by a third reviewer (S.S. or M.T.).

### Data collection process

A data extraction table comprising the first authors’ surname, publication date, study design, setting, sample size, age, female ratio, severity and phenotype of MS, scales about cognitive and sleep-related assessment, result and conclusion was designed. A review author contributed to fulfilling data extraction (H.R. or N.A. or S.M.), and another reviewer ascertained its accuracy (B.G. or A.N. and H.B.).

### Study risk of bias assessment

Two reviewers (B.G. and S.S-S.) independently appraised the risk of bias (RoB) and the risk of bias in the included articles using the Joanna Briggs Institute’s (JBI) critical appraisal tool for cross-sectional or case-control studies [39], and disagreements were resolved by discussion between the reviewers or by consulting with a third reviewer (S.S. or M.T.).

### Effect measures and synthesis methods

Any reported associations, including the difference between groups and/or correlations between sleep-related measures, and cognitive outcomes were presented in this systematic review. Due to the significant diversity regarding the assessed outcomes and reporting methods, conducting a meta-analysis was not possible; therefore, the outcomes of the studies were synthesized qualitatively.

## Results

### Study selection

**Fig. 1:**
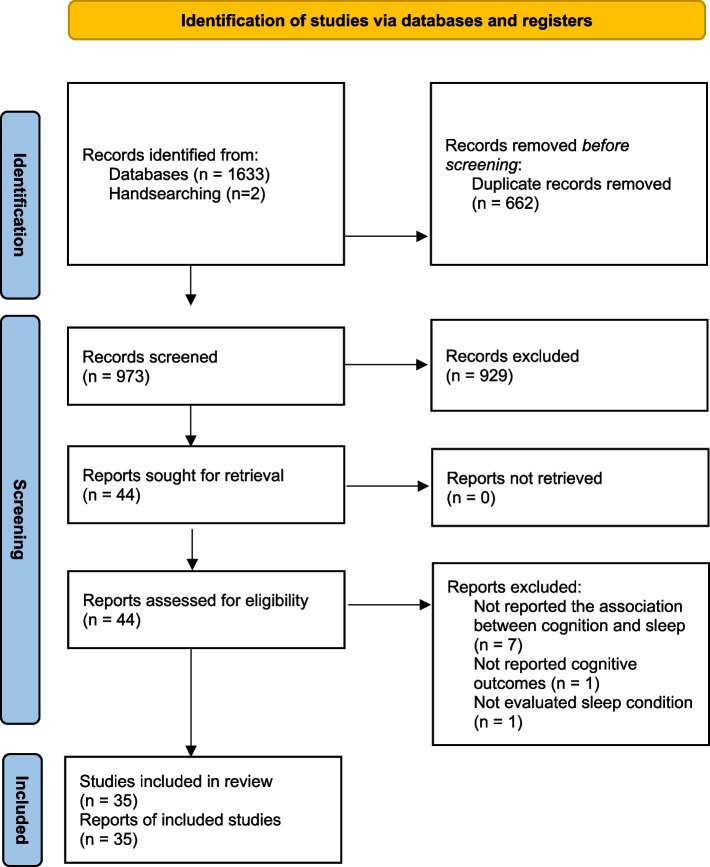
PRISMA flow diagram. *From*: Page MJ, McKenzie JE, Bossuyt PM, Boutron I, Hoffmann TC, Mulrow CD, et al. The PRISMA 2020 statement: an updated guideline for reporting systematic reviews. BMJ 2021;372:n71. doi: 10.1136/bmj.n71. For more information, visit: http://www.prisma-statement.org/

The systematic search identified 1635 records. After duplicate removal, 973 publications remained and screened for title/abstract; after that, 44 articles were selected for full-text review. Following the evaluation of selected studies for eligibility, nine articles were excluded due to the following reasons: seven studies didn’t report the association between cognition and sleep [40–46], one study didn’t report cognitive outcomes [47], and one study didn’t evaluate the sleep condition [48]. Finally, 35 articles met the eligibility criteria and were included. Figure 1 presents the details of the screening process.

### Risk of bias assessments

**Fig. 2:**
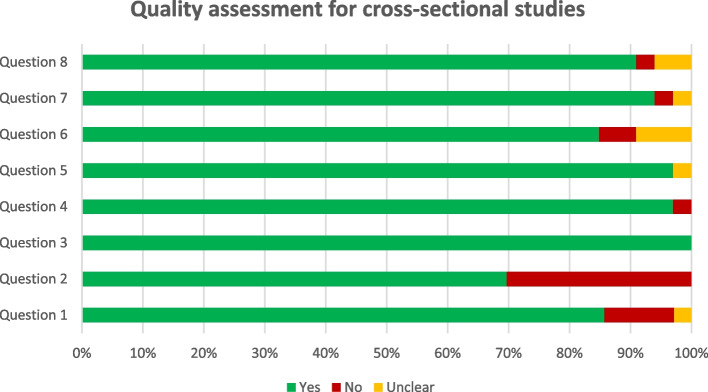
Summary of the quality assessment for cross-sectional studies. Question 1: Were the criteria for inclusion in the sample clearly defined? Question 2: Were the study subjects and the setting described in detail? Question 3: Was the exposure measured in a valid and reliable way? Question 4: Was objective, standard criteria used for measurement of the condition? Question 5: Were confounding factors identified? Question 6: Were strategies to deal with confounding factors stated? Question 7: Were the outcomes measured in a valid and reliable way? Question 8: Was appropriate statistical analysis used?

**Fig. 3:**
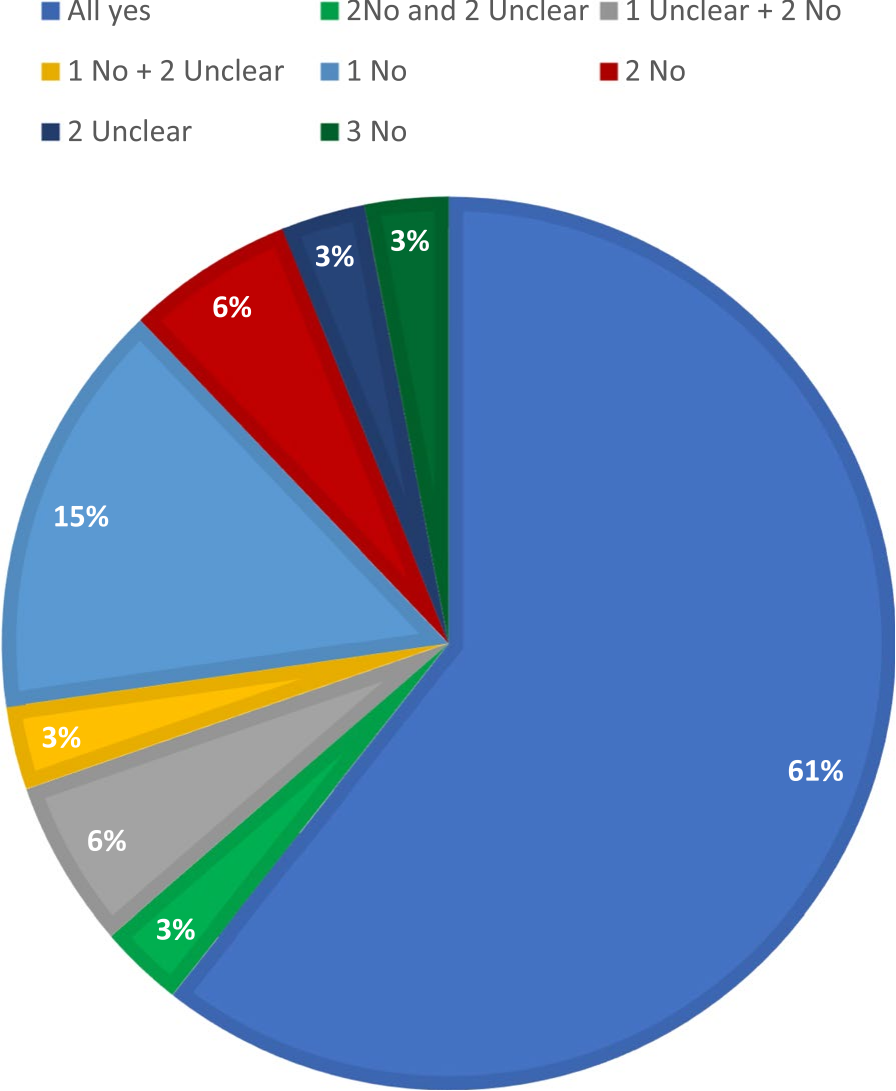
Articles quality regarding the answer to the questions

The results of RoB assessments are presented in Tables 1 and 2.; Figs. 2 and 3. Ten studies did not clarify the setting and six studies did not clearly define the inclusion/exclusion criteria. Generally, we did not find a considerable bias in a great number of studies, and none were excluded due to the high risk of bias. 61% of cross-sectional and all of the case-control studies were completely fitted to the JBI critical appraisal tools.

**Table 1:** Summary of the results of risk of bias assessments for cross-sectional studies

Study		1	2	3	4	5	6	7	8
(Aldughmi et al., 2016)	[49]	Yes	Yes	Yes	Yes	Yes	Yes	Yes	Yes
(Aristotelous et al., 2019)	[50]	Yes	Yes	Yes	Yes	Yes	Yes	Yes	Yes
(Beier et al., 2015)	[51]	Yes	Yes	Yes	Yes	Yes	Yes	Yes	Yes
(Berard et al., 2019)	[52]	No	No	Yes	Yes	Unclear	Unclear	Yes	Yes
(Braley et al., 2016)	[53]	Yes	Yes	Yes	Yes	Yes	Yes	Yes	Yes
(Braley et al., 2023)	[54]	Unclear	Yes	Yes	No	Yes	Yes	No	Yes
(Cederberg et al., 2020)	[55]	Yes	Yes	Yes	Yes	Yes	Yes	Yes	Yes
(Cederberg et al., 2022)	[56]	Yes	Yes	Yes	Yes	Yes	Yes	Yes	Yes
(Chen et al., 2023)	[57]	Yes	Yes	Yes	Yes	Yes	Yes	Yes	Yes
(Chinnadurai et al., 2018)	[58]	Yes	Yes	Yes	Yes	Yes	Yes	Yes	Yes
(Dubessy et al., 2021)	[59]	Yes	Yes	Yes	Yes	Yes	No	Yes	Yes
(Hare et al., 2019)	[60]	Yes	Yes	Yes	Yes	Yes	Yes	Yes	Yes
(Hughes et al., 2017)	[61]	Yes	Yes	Yes	Yes	Yes	Yes	Yes	Yes
(Hughes, Turner, et al., 2018)	[62]	Yes	Yes	Yes	Yes	Yes	Yes	Yes	Yes
(Lamis et al., 2018)	[63]	No	No	Yes	Yes	Yes	Yes	Yes	Yes
(Laslett et al., 2022)	[64]	Yes	Yes	Yes	Yes	Yes	Yes	Yes	Yes
(Lehmann et al., 2013)	[65]	No	No	Yes	Yes	Yes	Yes	Unclear	Yes
(Mackay et al., 2021)	[66]	Yes	Yes	Yes	Yes	Yes	Yes	Yes	Yes
(McNicholas et al., 2021)	[67]	Yes	Yes	Yes	Yes	Yes	Yes	Yes	Yes
(Odintsova & Kopchak, 2021)	[68]	Yes	No	Yes	Yes	Yes	Unclear	Yes	Unclear
(Opelt et al., 2023)	[69]	Yes	Yes	Yes	Yes	Yes	Yes	Yes	Yes
(Ozkul et al., 2020)	[70]	Yes	No	Yes	Yes	Yes	No	Yes	No
(Patel et al., 2017)	[71]	Yes	Yes	Yes	Yes	Yes	Unclear	Yes	Unclear
(Riccitelli et al., 2022)	[72]	Yes	No	Yes	Yes	Yes	Yes	Yes	Yes
(Sater et al., 2015)	[73]	Yes	Yes	Yes	Yes	Yes	Yes	Yes	Yes
(Schellaert et al., 2018)	[74]	Yes	Yes	Yes	Yes	Yes	Yes	Yes	Yes
(Shahrbanian et al., 2015)	[75]	Yes	No	Yes	Yes	Yes	Yes	Yes	Yes
(Siengsukon, Aldughmi, et al., 2018)	[76]	Yes	No	Yes	Yes	Yes	Yes	Yes	Yes
(Siengsukon, Alshehri, et al., 2018)	[77]	Yes	Yes	Yes	Yes	Yes	Yes	Yes	Yes
(Sumowski et al., 2021)	[78]	Yes	No	Yes	Yes	Yes	Yes	Yes	Yes
(van Geest et al., 2017)	[79]	No	No	Yes	Yes	Yes	Yes	Yes	Yes
(Valentine et al., 2023)	[80]	Yes	Yes	Yes	Yes	Yes	Yes	Yes	Yes
(Whibley et al., 2021)	[81]	Yes	Yes	Yes	Yes	Yes	Yes	Yes	Yes

Question 1: Were the criteria for inclusion in the sample clearly defined?

Question 2: Were the study subjects and the setting described in detail?

Question 3: Was the exposure measured in a valid and reliable way?

Question 4: Was objective, standard criteria used for measurement of the condition?

Question 5: Were confounding factors identified?

Question 6: Were strategies to deal with confounding factors stated?

Question 7: Were the outcomes measured in a valid and reliable way?

Question 8: Was appropriate statistical analysis used?

**Table 2:** summary of the results of risk of bias assessments for case-control studies

**Study**		**1**	**2**	**3**	**4**	**5**	**6**	**7**	**8**	**9**	**10**
**(Borragán et al., 2018)**	[82]	Yes	Yes	Yes	Yes	Yes	Yes	Yes	Yes	Yes	Yes
**(Terauchi et al., 2023)**	[83]	Yes	Yes	Yes	Yes	Yes	Yes	Yes	Yes	Yes	Yes

Question 1: Were the groups comparable other than the presence of disease in cases or the absence of disease in controls?

Question 2: Were cases and controls matched appropriately?

Question 3: Were the same criteria used for identification of cases and controls?

Question 4: Was exposure measured in a standard, valid, and reliable way?

Question 5: Was exposure measured in the same way for cases and controls?

Question 6: Were confounding factors identified?

Question 7: Were strategies to deal with confounding factors stated?

Question 8: Were outcomes assessed in a standard, valid, and reliable way for cases and controls?

Question 9: Was the exposure period of interest long enough to be meaningful?

Question 10: Was appropriate statistical analysis used?

### Study characteristics and results of individual studies

The design of 29 studies was cross-sectional, three studies were longitudinal, one article was a pilot study, and two studies were case-control. Most studies were conducted in the United States (14 studies). Sample sizes varied between nine and 1717 participants, with a total number of 5321. The age of participants ranged from 32.4 to 59.7 years. The female ratio was between 54.5 and 100%.

The severity of MS was mostly reported by the Expanded Disability Status Scale (EDSS) score, although four studies didn’t report the severity of MS and some of the studies reported the severity of MS by other scales like the Multiple Sclerosis Functional Composite (MSFC), and Patient Determined Disease Steps (PDDS). MS phenotype in five studies was RRMS; in three studies it was not reported, and in the remaining 27 studies, a mixture of different phenotypes was investigated.

The studies used different kinds of assessment scales, both for cognition and sleep. Sleep-related assessment scales include but are not limited to actinography, polysomnography (PSG), Pittsburgh Sleep Quality Index (PSQI), Epworth Sleepiness Scale (ESS), and Insomnia Severity Index (ISI). Cognition assessment scales also included but not limited to the Paced Auditory Serial Addition Test (PASAT), California Verbal Learning Test (CVLT), Brief Visuospatial Memory Test (BVMT), Symbol Digit Modalities Test (SDMT), Judgement of Line Orientation Test (JLO), Controlled Oral Word Association Test (COWAT), Hopkins Verbal Learning Test (HVLT), Cambridge Neuropsychological Test Automated Battery (CATNAB), Letter Digit Substitution Task (LDST), and Delis-Kaplan Executive Function System–Sorting Test (D-KEFS-S).

Detailed characteristics and findings of the included studies are shown in Table 3. Considering the substantial diversity in both sleep and cognitive evaluation scales, quantitative synthesis was not feasible. Meta-synthesis of the most common combinations of sleep and cognitive evaluations are presented in Table 4.

**Table 3:** Summary of the characteristics and findings of the included studies

Study		Study design	Setting	Sample size	Age	Female ratio	Severity of MS	disease phenotypes	Cognitive assessment scale	Sleep assessment scale	Results	Conclusion
[49]	**(Aldughmi et al.**,** 2016)**	cross-sectional	MS clinic at the University of Kansas Medical Centre, US	51	47 ± 10.1	43 (84.3%)	1.8 ± 1.6	RRMS (90.2%), SPMS (9.8%)	Cognitive fatigability (CPT-RSV)	PSQI, Actigraphy (SE, TST, TST in bed, WASO, NoA)	The RSV score was significantly associated with only the daytime dysfunction component of the PSQI (r: 0.303; p-value: 0.030), as well as sleep efficiency (r: 0.342; p-value: 0.015), and wake after sleep onset in the actigraph (r: 0.294; p-value: 0.039); however, these associations were no longer significant after controlling for depression (sleep efficiency: r: -0.232; p-value: 0.105; wake after sleep onset: r: 0.185; p-value: 0.199)	Poor sleep quality may be related to decreased cognitive performance, which is mediated by depression.
[50]	**(Aristotelous et al.**,** 2019)**	cross-sectional	Cyprus Institute of Neurology and Genetics	51	38.4 ± 7.1	30 (58.8%)	2.38 ± 1.04	RRMS (100%)	PASAT	PSQI	A significant association between PSQI and PASAT scores in linear regression was observed (R: 0.334; p-value: 0.017)	Poorer sleep quality is associated with lower cognitive performance.
[51]	**(Beier et al.**,** 2015)**	longitudinal	Community (US)	407	52.95 ± 10.67	338 (83%)	≤ 4.0: (31.4%), 4.5–6.5: (48.6%), ≥ 7.0: (20%)	RRMS (57.2%), SPMS (20.9%), PPMS (11.1%), PRMS (8.6%)	Neuro-QOL-EF, Neuro-QOL-GC	PROMIS Sleep Disturbance And Sleep-related Impairment (SRI)	For general cognitive concerns, there was a small, negative correlation with sleep disturbance (r: -0.29; p-value < 0.05) and a medium, negative correlation with sleep-related impairment (r: -0.39; p-value < 0.05)	Day-time sleep-related impairment and sleep disturbance are significantly correlated with self-reported cognitive function.
[52]	**(Berard et al.**,** 2019)**	cross-sectional	MS Clinic at the Ottawa Hospital, Canada	58	-	-	-	-	PASAT	PSQI	Sleep quality was the greatest and only significant independent predictor of cognitive function (r: 0.433; p-value: 0.001)	Sleep quality is the most significant predictor of cognitive fatigue.
[82]	**(Borragán et al.**,** 2018)**	case-control	Hôpital Erasme, Anderlecht, Belgium	9	42.1 ± 7.1	-	1.65 ± 0.77	RRMS (100%)	self-reported cognitive fatigue, TloadDback	Polysomnography (TST)	A significant between-group effect (Adjusted R squared > 29% variance; p-value < 0.05) suggesting that MS patients who were sleeping more experienced a higher triggering of cognitive function during the TloadDback	Total sleeping time discloses a significant effect on cognitive fatigue.
[53]	**(Braley et al.**,** 2016)**	Cross-sectional	University of Michigan MS Center, US	38	48.3 ± 10.12	21 (55.3%)	3.39 ± 1.58	RRMS (65.8%), SPMS (21.1%), PPMS (13.2%)	CVLT-II, BVMT-R, SDMT, PASAT, JLO, COWAT, D-KEFS-S	Polysomnography (TST, SE, SL, WASO, TAI, REM, RDI, ODI, MOS), ESS	Sleepiness was only associated with CVLT-II discriminability index (r: 0.41; p-value: 0.03). A higher respiratory disturbance index was associated with lower PASAT (r: −0.52; p-value: 0.001) and SDMT scores (r: −0.39; p-value: 0.02). TAI was inversely associated with PASAT (r: −0.33; p-value: 0.04), SDMT (r: −0.38; p-value: 0.02), and CVLT-II Discriminability Index scores (r: −0.51; p-value: 0.001). TST positively correlated with CVLT-II Discriminability Index scores (r: 0.40; p-value: 0.01). After adjustments for age, disease duration, years of education, and depressive symptoms, significant associations were revealed between oxygen desaturation index and PASAT-3 (β: -0.38; p-value: 0.02); BVMT – Delayed (β: −0.47; p-value < 0.01). Total arousal index meaningfully related with CVLT-II Discriminability (β: -0.53, p-value < 0.001). COWAT and D-KEFS-S scores were not correlated with any sleep measures	OSA and sleep disturbance are significantly associated with diminished visual memory, verbal memory, executive function, attention, processing speed, and working memory.
[54]	**(Braley et al.**,** 2023)**	Longitudinal	The Nurse’s Health Study (NHS II Cohort)	524	59.0 ± 4.3	524 (100%)	-	-	Self-reported recent situational difficulty in remembering and subjective cognitive function	STOP-Bang, OSA diagnosis, sleep quality and Sleepiness	Insomnia mediated 5.4–15.1% of the total effect between MS and conversations/plots, and memory impairment, while sleepiness mediated 8.6–12.3% of the total effect for these outcomes. OSA significantly accounted for 34% of the total effect between MS and following instructions. After adjustment for age, race, smoking, marital status, and employment, MS symptomatic sleepiness represented difficulty in following spoken instruction (OR: 1.1; 95%CI: 0.87, 1.2), difficulty in following conversions or a plot (OR: 1.0; 95%CI: 0.84, 1.2) and difficulty in finding your way around familiar streets (OR: 0.79; 95%CI: 0.55, 1.0)	Patients with OSA, insomnia, or sleepiness may experience increased subjective cognitive dysfunction.
[55]	**(Cederberg et al.**,** 2020)**	Cross-sectional	North American Research Committee on MS	275	59.7 ± 10.1	223 (81.1%)	PDDS: 3.0 [5]	RRMS (65.8%), PMS (33.1%), Unknown (1.1%)	MSNQ	IRLS, PSQI	Patients presenting RLS reported significantly worse perceived cognitive impairment (p-value: 0.015). The inclusion of sleep quality attenuated the relationship between RLS severity and perceived cognitive impairment (β: 0.126; p-value > 0.05). More severe perceived cognitive impairment was significantly associated with worse sleep quality (r: 0.44)	Sleep impairment may be an intermediary factor in the association between RLS severity and cognitive impairment.
[56]	**(Cederberg et al.**,** 2022)**	Exploratory Pilot Study	University of Alabama at Birmingham	22	51.6 ± 12.0	17 (77.3%)	3.5 ± 2.6	RRMS (72.7%), PMS (27.3%)	SDMT, CVLT-II, BVMT-R	IRLS, RLS-6	Worse IRLS total severity was associated with lower SDMT (ρ: −0.42; p-value < 0.05), worse CVLT-II (ρ: −0.63; p-value < 0.01), and worse BVMT-R (ρ: −0.61; p-value < 0.01). Worse RLS severity at falling asleep was associated with worse CVLT-II (ρ: -0.45; p-value < 0.05). There was no association between RLS severity at falling asleep and SDMT. Worse RLS severity during the day while active was associated with lower SDMT (ρ: −0.58; p-value < 0.01), CVLT-II, (ρ: −0.52; p-value: 0.05), BVMT-R (ρ: -0.60; p-value < 0.01).	More severe RLS might be associated with worse cognitive function, particularly slower processing speed, and more memory difficulties.
[57]	**(Chen et al.**,** 2023)**	Cross-sectional	National MS Society and Kessler Foundation websites	45	41.69 ± 13.39	41 (91.1%)	0–2 (11.1%) 2.5 (26.67%) 3.0 (31.11%) 3.5–4.5 (24.45%) > 4.5 (6.66%)	RRMS (86.67%), PPMS (6.67%), SPMS (4.44%), Not sure (2.22%)	Smartphone-based TMT-B completion time, self-report cognitive function	Self-report sleep time and difficulty in sleeping	With considering possible confounders, prior night sleep duration was not significantly correlated to TMT-B performance (coefficient: −0.12; p-value: 0.104). Self-report sleep difficulties were significantly correlated to mTMT-B performance (coefficient: 0.08; p-value: 0.045). Regarding self-reported cognitive dysfunction, the correlations were not significant (p-value > 0.05)	Prior night sleep conditions, do not seem to significantly affect cognitive function.
[58]	**(Chinnadurai et al.**,** 2018)**	Cross-sectional	Institute of Neurology, Madras Medical College, India	113	32.43 ± 9.90	74 (65.49%)	2.91 ± 1.02	RRMS (55.75%), SPMS (26.55%), PPMS (17.70%)	MFIS-C, Stroop test, mSDMT, SAT	Polysomnography (N1, N2, N3, REM, SOL, SE, WASO, REI, PLMI)	The SAT fatigue scores correlated with SE (r: -0.240, p-value: 0.01), SOL (r: 0.278, p-value: 0.003), and percentage of N2 sleep (r: − 0.302, p-value: 0.001). mSDMT fatigue scores correlated with SE (r: − 0.309, p-value: 0.001), SOL (r: 0.411, p-value < 0.001), percentage of N1 sleep (r: 0.275, p-value: 0.003), percentage of N2 sleep (r: − 0.254, p-value: 0.007), and WASO (r: 0.374, p-value < 0.001). MFIS-C showed a significant correlation with SE (r: − 0.328, p-value: 0.01), SOL (r: 0.491, p-value < 0.001), percentage of N1 sleep (r: 0.506, p-value < 0.001), percentage of N2 sleep (r: − 0.444, p-value < 0.001), percentage of N3 sleep (r: − 0.429, p-value < 0.001), percentage of REM sleep (r: − 0.253, p-value < 0.001) and WASO (r: 0.506, p-value < 0.001). MFIS-C scores did not correlate with either REI (r: − 0.009, p-value: 0.922) or PLMI (r: − 0.051, p-value: 0.593).	A significant relationship exists between sleep impairment and cognitive fatigue.
[59]	**(Dubessy et al.**,** 2021)**	Cross-sectional	Pitie Salpetriere Hospital in Paris and Purpan Hospital in Toulouse, France	44	41.7 (38.5–48.6)	28 (64%)	2.0 (1.5-3.0)	RRMS (86%), PPMS (14%)	MDRS, PASAT	Polysomnography (TST, SE, WASO, SOL, N1, N2, N3, REM, TAI, PLMI, AHI), ESS, MSLT	Cognitive performances were similar between patients with and without abnormal polysomnography results meeting the diagnostic criteria for either narcolepsy or hypersomnia.	There was no significant link between central hypersomnia and cognitive performance.
[60]	**(Hare et al.**,** 2019)**	Cross-sectional	Community, Toronto, Canada.	114	44.0 ± 11.59	111 (93.3%)	current MS symptom severity: 44.50 ± 30.70	RRMS (77.7%), SPMS (11.7%), PPMS (8.7%), PRMS (1.9%)	MFIS-CF, RRQ	ISI	The relationship between insomnia symptoms and cognitive fatigue was significant (β: 0.41, p-value: 0.008). The relationship between ISI and MFIS-CF was significant, which is only mediated by the fatigue catastrophizing.	Fatigue catastrophizing mediated the relationship between insomnia symptom severity and cognitive fatigue.
[61]	**(Hughes et al.**,** 2017)**	Cross-sectional	two VA Medical Centers, US	121	52.1 ± 9.1	66 (54.5%)	mild (36.4%), moderate (31.4%), severe (32.2%)	RRMS (64.5%) PMS (34.5%)	CVLT-II, PASAT, COWAT, VSCWT, PDQ	PSQI	There was no significant difference between cognitively impaired and unimpaired groups regarding PSQI (p-value: 0.07). With controlling for demographic and clinical covariates, the PSQI subscales are most commonly associated with perceived cognitive impairment (all *P* < 0.05).	Self-reported sleep is significantly and independently related to perceived but not objective cognitive impairment in MS.
[62]	**(Hughes**,** Turner**, et al.,** 2018)**	Longitudinal	US	163	52.2 ± 10.1	142 (87.1%)	PDDS: 12.0 ± 9.0	RRMS (55.8%), SPMS (20.9%), PPMS (17.2%), PRMS (4.9%)	Neuro-QoL Applied Cognition–General Concerns Short Form Version 1	MOS-SS	A significant direct and indirect effect of self-reported sleep problems on perceived cognitive dysfunction via fatigue impact was demonstrated (direct effect: β: − 0.04, p-value: 0.02; indirect effect: β: − 0.06, p-value < 0.001).	Sleep problems may account for some of the perceived cognitive dysfunction in MS.
[63]	**(Lamis et al.**,** 2018)**	Cross-sectional	An outpatient, university-based MS Clinic, US.	77	51.12 ± 9.6	64 (83%)	-	RRMS (100%)	PDQ	PSQI	Higher levels of perceived cognitive deficits were associated with poorer sleep quality (B: 0.13; 95%CI: 0.07–0.19). The total effect of perceived cognitive deficits on depressive symptoms was significantly mediated by sleep quality (aB: 0.106; 95%CI: 0.026–0.215).	Poor sleep quality is mediated by the correlation between depression and cognitive deficits in MS.
[64]	**(Laslett et al.**,** 2022)**	Cross-sectional	Australian MS Longitudinal Study	1717	57.7 ± 11.3	136 (80%)	PDDS score 0: 23% 1–2: 23% 3–5: 35% ≥ 6: 19%	Relapse-onset (76%), Progressive-onset (13%)	MSSymS	PSQI, ESS, IRLS	Cognitive symptoms were associated with poorer sleep quality (p-value < 0.001), even in multivariate analyses (β: 0.21; 95%CI: 0.10 to 0.33)	The “Fatigue and cognition” cluster is independently associated with sleep quality.
[65]	**(Lehmann et al.**,** 2013)**	Cross-sectional	MS clinics in Germany and the Netherlands	42	46.9 ± 7.7, 47.3 ± 8.1	-	3.14 ± 1.52, 3.28 ± 2.28	RRMS (100%)	n-back-hits, n-back-RT	PSQI	MS patients with sleep disturbances show cognitive decline during tasks of sustained attention, while there was no difference between groups regarding age, education, premorbid intelligence, and EDSS.	Patients with self-reported sleep problems showed cognitive fatigability, which could be improved with a restorative rest period.
[66]	**(Mackay et al.**,** 2021)**	Cross-sectional	academic MS clinic in London, Canada	53	44.23 ± 11.19	37 (69.81%)	Median: 2.00	RRMS (88.68%), SPMS (9.43%), PPMS (1.89%)	PASAT, MFIS-CF, SDMT, ZVT	PSQI	The cognitive scale of the MFIS demonstrated a statistically significant relationship with the total PSQI (r: 0.33; p-value: 0.02); however, PASAT, SDMT, and ZVT did not demonstrate. Subjective sleep quality (r: 0.39), sleep latency (r:0.38), and daytime dysfunction (r: 0.33) are associated with the cognitive scale of the MFIS (p-values < 0.05).	Although sleep quality correlated with subjective CF, this study did not find depression mediates this relationship.
[67]	**(McNicholas et al.**,** 2021)**	cross-sectional and longitudinal study	a University Hospital MS Clinic, Dublin, Ireland	23	45.3	17 (74%)	1.9	RRMS (87%), SPMS (13%)	SDMT, CVLT, BVMT, PASAT-3, MFIS	OSA (AHI)	No correlation was seen between any cognitive parameter and the AHI (p-values > 0.05). A significant improvement with OSA treatment was only seen in CVLT-2 (before-after mean difference: 9.5 ± 7.9; p-value: 0.02). A significant correlation was seen between CVLT-2 and minimum SpO2 (r: 0.527; p-value: 0.01).	OSA treatment may improve cognition, specifically verbal memory in MS.
[68]	**(Odintsova & Kopchak**,** 2021)**	Cross-sectional	Kyiv, Ukraine	105	41.8 ± 10.7	80 (76.19%)	3.5 ± 1.6	RRMS (100%)	MoCA	PSQI	No difference was found in patients with and without sleep disorders according to the MoCA score (p-value: 0.95). No correlations were observed (r: -0.25, p-value: 0.10).	This study does not confirm the correlation between sleep disorders and cognitive impairment.
[69]	**(Opelt et al.**,** 2023)**	Cross-sectional	Community-based sample, US.	90	48.04 ± 14.25	73 (81%)	PDDS: 1.98 ± 1.87	RRMS (76%), SPMS (14%), PPMS (10%)	MFIS-CF MSNQ	PSQI and Actigraphy discrepancies (SL, TST, SE)	Clinically significant cognitive symptoms evidenced greater PSQI-actigraphy discrepancies in SL (p-value: 0.003) and TST (p-value: 0.01).	Discrepancies between self-reported and actigraphy sleep measures are associated with cognitive outcomes in MS.
[70]	**(Ozkul et al.**,** 2020)**	Cross-sectional	Gazi University, Ankara, Turkey	112	34	44 (77.2%), 43 (78.2%)	2, 1	RRMS (80.7%) PPMS (19.3%) AND RRMS (90.9%) PPMS (9.1%)	SPART, SRT, SDMT, PASAT	PSQI	PSQI scores were similar between cognitively impaired and cognitively normal groups (p-value: 0.359). Correlations between PSQI and SPARTT (r: −0.199), SPARTD (r: −0.145), SRT-ST (r: −0.046), SRT-LT (r: 0.016), WLG (r: 0.005), SDMT (r: −0.058), and PASAT (r: −0.141) were not significant (p-values > 0.001).	This study did not find a relationship between cognitive domains and sleep quality.
[71]	**(Patel et al.**,** 2017)**	Cross-sectional	Outpatient neurology clinics Canada	102	44.61 ± 10.01	70 (68.6%)	2.69 ± 2.03	RRMS (69.6%), SPMS (22.5%), PPMS (7.8%)	SDMT, c-SDMT	ESS	Patients with excessive daytime sleepiness were significantly slower on the distracter c-SDMT (t: −2.689; p: 0.01) which equated with greater impairment (68.8% vs. 36.1%; p-value: 0.029). , but not in c-SDMT without distracters (t: 0.642; p-value: 0.524) and traditional SDMT (t: 1.171; p-value: 0.247).	Excessive daytime sleepiness adversely influences processing speed in the presence of real-world distracters.
[72]	**(Riccitelli et al.**,** 2022)**	Prospective cross-sectional monocentric	Neuro Center in Lugano, Southern Switzerland	80	47.16 ± 10.18	57 (71.5%)	2.65 ± 1.38	RRMS (92.5%), PMS (7.5%)	BRB-N	PSQI, ESS, Polysomnography (TST, SE, SOL, TAI, WASO, RDI, PLMI, ODI, NoA, N1, N2, N3, REM)	Worse performances at global (r: 0.33, p-value: 0.003), memory (r: 0.30; p-value: 0.008), and attention (r: 0.31; p-value: 0.006) cognitive domains were correlated with reduced sleep efficacy and negatively associated with wake after sleep onset (r: −0.33; p-value: 0.004; r: −0.35; p-value: 0.002; r: −0.24, p-value: 0.034, respectively). Poor attention performances were associated with reduced REM sleep (r: 0.26; p-value: 0.022). Other correlations were not significant (p-value > 0.05).	Low sleep efficiency, nocturnal wake, and reduced REM sleep might affect cognitive abilities in MS.
[73]	**(Sater et al.**,** 2015)**	Cross-sectional	USA	32	45.66 ± 9.15	24 (75.0%)	2.72 ± 1.75	-	MFIS-Cognitive, NeuroTrax tests	Polysomnography (N1, N2, N3, REM, SOL, SE, WASO, AHI, EDI, PLMI), MSLT, ESS	Sleep efficacy correlated with the global score (r: 0.359; p-value: 0.043), executive function (r:0.405; p-value: 0.021), and information processing subscales (r: 0.384; p-value: 0.030). WASO negatively correlated with the verbal function subscale (r: -0.363; p-value: 0.041). SOL negatively correlated with executive function and attention. The AHI, TAI, and OSA did not correlate with cognitive measures. The duration of daytime sleep latency on MSLT negatively correlated with the global cognition score. ESS mildly correlated with the cognitive component of the MFIS (r: 0.406; p-value: 0.021). MS patients with OSA did not differ from those without OSA in the global score or any of the subscales (p-values > 0.05)	Poor sleep efficiency may contribute to reduced cognitive function.
[83]	**(Terauchi et al.**,** 2023)**	Case-control study	Iwate Medical University, Japan	67	42.9 ± 9.3	72 (73.46%)	4.9 ± 2.5	RRMS (80.6%), PPMS (1.5%), SPMS (17.9%)	SDMT, PASAT, MMSE	Out of Center Sleep Testing (REI)	REI < 5 and ≥ 5 groups were not different regarding SDMT, PASAT, and MMSE scores (p-values > 0.05).	Sleep apnea can facilitate cognitive decline in people living with MS.
[74]	**(Schellaert et al.**,** 2018)**	Cross-sectional	The University Department of Neurology in Montpellier, France	57	46.8 ± 12.0	36 (63%)	4.79 ± 2.28	RRMS (36.8%), PRMS (28.1%), SPMS (35.1%)	The cognitive dimension of EMIF-SEP	ISI, DBAS-16, SRBQ, PSAS	Patients with insomnia disorder described higher cognitive (p-value < 0.001) and somatic (p-value: 0.001) manifestations of arousal.	Insomnia disorder comorbid to MS is associated with the cognitive factors in MS.
[75]	**(Shahrbanian et al.**,** 2015)**	Cross-sectional	Three major MS clinics in greater Montreal, Canada	188	43.0 ± 10.0	139 (73.9%)	2.4 ± 2	RRMS (51.6%), SPMS (3.7%), PPMS (4.3%), PRMS (1.6%), CIS (4.8%)	PDQ	PSQI-R	Cognitive deficits were significantly correlated with sleep disorders (r: 0.03; p-value < 0.0001).	Cognitive deficits are linked with sleep disorders.
[76]	**(Catherine F. Siengsukon**, et al.,** 2018)**	Cross-sectional	MS clinic at the University of Kansas Medical Center, USA	40	50.3 ± 11.6	36 (90.0%)	MSFC: Poor sleep: 0.792 ± 1.9 Good sleep: 0.69 ± 0.796	RRMS (82.5%), PMS (17.5%)	SDMT, PASAT, HVLT, BVMT, Stroop	PSQI	Patients with good sleep quality performed significantly better on BVMT (effect size: 0.35; p-value: 0.025), but not in HVLT (effect size: 0.11; p-value: 0.512), PASAT (effect size: 0.22; p-value: 0.168), SDMT (effect size: 0.29; p-value: 0.073), Stroop (effect size: 0.16; p-value: 0.308),	Visuospatial memory is lower in individuals with poor sleep quality
[77]	**(Catherine F Siengsukon**, et al.,** 2018)**	Cross-sectional	MS specialty clinic at the University of Kansas Medical Center	66	47.80 ± 10.74	57 (86%)	-	RRMS: 55 (83.3%) SPMS: 11 (16.7%)	MFIS-CF	PSQI, ESS, TST	Patients with poor sleep quality/high sleep time variability had a significantly higher level of cognitive fatigue (p: 0.002).	Bad sleep is linked to cognitive function.
[78]3	**(Sumowski et al.**,** 2021)**	Cross-sectional	The Reserve against Disability in Early MS (RADIEMS) Cohort, New York, US	184	34.3 ± 7.4	123 (66.8%)	1.0 (0.0-1.5)	RRMS (89.1%), CIS (10.9%)	CANTAB PAL, BVMT-R, SRT, V-PAL, SDMT, Stroop, Pattern Comparison, Decision Speed	ISI	Disturbed sleep was specifically related to CANTAB PAL and V-PAL, with controlling for multiple confounders (p-values < 0.001). The association between SDMT and sleep disturbance was significant, only when controlling for age and sex. The association between Stroop and sleep disturbance was significant when controlling for age and sex as well as age, sex, mood, fatigue, medications, and BMI.	This study connects patient-reported sleep disturbance specifically to poor memory in early MS.
[79]	**(van Geest et al.**,** 2017)**	Cross-sectional	Amsterdam, The Netherlands	71	45.7 ± 8.3	47 (66.2%)	Normal sleep: 3.50 Sleep disturbed: 4.00	RRMS (73.2%), PMS (25.4%)	VLGT, LLT, LDST, DS-F, DS-B, WLG	AIS	52% of sleep-disturbed patients were categorized as cognitively impaired versus 29% of normal sleeping patients (p-value: 0.060). Sleep-disturbed patients reported higher levels of subjective cognitive problems (p-value: 0.023). No significant relationship was found between AIS score and overall objective cognitive functioning or individual neuropsychological test scores	Sleep disturbances, as measured with the AIS, do not directly relate to objective cognitive functioning, but rather to subjective cognitive problems.
[80]	**(Valentine et al.**,** 2023)**	Cross-sectional	University of Michigan’s MS Clinic and Multidisciplinary MS Fatigue and Sleep Clinic, and through fliers, a public website, and social media advertisements, the US	131	48.28 ± 9.42	91 (69.5%)	0–4.0 (42.7%); 4.5–6.5 (55.7%); 7.0–9.5 (1.6%)	RRMS (80.2%), PPMS (5.3%), SPMS (11.5%), PRMS (0.8%), Unknown (2.3%)	SDMT, PASAT, CVLT-II, BVMT-R, JLO, COWAT, Trails A and B, NHPT	Polysomnography indices (Apnea–hypopnea, Oxygen desaturation index, Mean O2 saturation, Minimum O2 saturation, Obstructive apnea, central apnea, hypopnea, Periodic limb movement, N1%, N2%, N3%, R%, wake after sleep onset, sleep efficiency, sleep latency, TST)	Apnea severity measures were associated with SDMT, BVMT-R Total and Delayed, Trails, and NHPT (p-value⩽0.011). Sleep macrostructure measures showed stronger associations with CVLT-II and BVMT-R Total) (p-value⩽0.011), with and without controlling for confounders.	Pathological sleep is differentially associated with worse cognitive performance.
[81]	**(Whibley et al.**,** 2021)**	Cross-sectional	University of Michigan, USA	49	47.6 ± 10.0	61%	3.0	RRMS (67%), SPMS (23%), PPMS (10%)	CVLT-II, PASAT, SDMT, BVMT-R, JLO, COWAT, D-KEFS	ESS, PSQI Actigraphy (RID, TST, SOL, WASO, SE)	A higher composite sleep score was associated with a lower JLO score (B: −0.82; p-value: 0.02). short or long sleep duration was associated with a higher BVMT-delayed score, and poor sleep quality was associated with higher COWAT scores (p-value: 0.04). There was a positive association between D-KEFS and sleep rhythmicity (p-value: 0.02). A higher composite sleep score was associated with a lower D-KEFS score (p-value: 0.048).	Associations were observed between measures of cognitive function and sleep fragmentation, duration, quality, rhythmicity, and composite score.

*Abbreviations*: *AHI *Apnea-hypopnea index, *AIS *AAthens Insomnia Scale, *AT *Attention, *BRB* Brief Repeatable Battery, *BVMT* Brief Visuospatial Memory Test, *BVMT-R* Brief Visuospatial Memory Test–Revised, *CANTAB* Cambridge Neuropsychological Test Automated Battery, *CIS* Clinically isolated syndrome, *COWAT* Controlled Oral Word Association Test, *CPT-RSV* Continuous Performance Test–Response Speed Variability, *CVLT-II* California Verbal Learning Test–II, *DBAS-16* Brief version of the Dysfunctional Beliefs and Attitudes about Sleep, *D-KEFS-S* Delis-Kaplan Executive Function System–Sorting Test, *DS-B* Digit span–backward, *DS-F* Digit Span–forward, *EF* Executive Function, *EMIF-SEP* Fatigue Impact Scale for multiple sclerosis, *ESS* Epworth Sleepiness Scale, *FSMC* Fatigue Scale for Motor and Cognitive Functions, *GC* Global cognition, *HVLT* Hopkins Verbal Learning Test, *ID* Insomnia disorder, *IRLS* International Restless Legs Syndrome, *ISI* Insomnia Severity Index, *JLO* Judgement of Line Orientation Test, *LDST* Letter Digit Substitution Task, *LLT* Location Learning Test, *MACFIMS* Minimal Assessment of Cognitive Function in Multiple Sclerosis, *MDRS* Mattis Dementia Rating Scale, *MFIS-CF* Modified Fatigue Impact Scale–Cognitive Function, *MoCA* Montreal Cognitive Assessment, *MOS* Minimal Oxygen Saturation, *MOS-CFS* Medical Outcome Study–Cognitive Function Scale, *MOS-SS* Medical Outcome Study–Sleep Scale, *MS* Multiple sclerosis, *mSDMT* Modified Symbol Digit Modalities Test, *MSL* Mean daytime sleep latency, *MSLT* Multiple sleep latency test, *MSNQ* Multiple Sclerosis Neuropsychological Questionnaire, *MSSymS* MS Symptom Score, *MVb* Verbal memory, *MVs* Visual memory, *N1* Non-REM1 Sleep Stage, *N2* Non-REM2 Sleep Stage, *N3* Non-REM3 Sleep Stage, *NARCOMS* North American Research Committee on Multiple Sclerosis patient registry, *Neuro-QoL-AC* Neuro-QoL–Applied Cognition, *Neuro-QOL-EF* Neuro-QoL– executive functioning, *Neuro-QOL-GC* Neuro-QoL– general cognition, *NoA* Number of Awakenings, *ODI* Oxygen Desaturation Index, *PASAT* Paced Auditory Serial Addition Test, *PDQ* Perceived Deficits Questionnaire, *PMS* Progressive MS, *PLMI* Periodic Limb Movement Index, *PROMIS* Patient-Reported Outcomes Information System, *PS* Processing speed, *PSAS* Pre-Sleep Arousal Scale, *PSQI* Pittsburgh Sleep Quality Index, *PSQI-R* Pittsburgh Sleep Quality Index–Rasch Analysis, *RDI* Respiratory Disturbance Index, *REI* Respiratory Event Index, *RID* Rest Interval Duration, *RLS-6* Restless Legs Syndrome-6 Scale, *RRMS* Relapsing-remitting MS, *SAT* Serial Addition Test, *SCL-90* Symptom Checklist-90, *SDMT* Symbol Digit Modalities Test, *SE* Sleep efficiency, *SL* Sleep latency, *SOL* Sleep-onset latency, *SPART* Spatial Recall Test, *SRBQ* Sleep-Related Behaviors Questionnaire, *SRT* Selective Reminding Test, *STOP-BANG* (Snoring, Tiredness, Observed apneas, high blood Pressure, BMI, Age, Neck circumference, Gender), *TAI* Total Arousal Index, *TMT* Trail-Making Test, *TST* Total sleep time, *V-PAL* Verbal Paired Associate Learning, *VLGT* Verbale Leer en Geheugen Test, *VSCWT* Victoria Stroop Color-Word Test, *WAIS* Wechsler Adult Intelligence Scale, *WASO* Wake after sleep onset, *WLG* World List Generation, *ZVT  *Der Zahlen Verbindungs Test, *TloadDback* Working memory updating task, *RRQ* Rumination and Reflection Questionnaire

**Table 4:** Associations of common cognition scales with common sleep-related scales

Cognitive assessment scale	Sleep-related assessment scale	Effect size data	Study	
PASAT	PSQI	R: 0.334; p-value: 0.017	[50]	(Aristotelous et al., 2019)
		R: 0.433; p-value: 0.001	[52]	(Berard et al., 2019)
		Effect size: 0.22; p-value: 0.168	[76]	(Catherine F. Siengsukon, et al., 2018)
		R: −0.141; p-value > 0.001	[70]	(Ozkul et al., 2020)
		B: 0.01; t: 0.08; p-value: 0.94	[61]	(Hughes et al., 2017)
		Not reported but not significant	[81]	(Whibley et al., 2021)
		R: 0.03; p-value > 0.05	[66]	(Mackay et al., 2021)
SDMT	PSQI	Not reported but not significant	[81]	(Whibley et al., 2021)
		Effect size: 0.29; p-value: 0.073	[76]	(Catherine F. Siengsukon, et al., 2018)
		R: −0.058; p-value > 0.001	[70]	(Ozkul et al., 2020)
		R: -0.19; p-value > 0.05	[66]	(Mackay et al., 2021)
SDMT	ESS	Not reported but not significant	[53]	(Braley et al., 2016)
		t: 0.642; p-value: 0.524	[71]	(Patel et al., 2017)

### PSQI and cognitive outcomes

PSQI is a self-report questionnaire that considers sleep quality and disturbances over one month [84]. PSQI was the most common sleep assessment instrument in the included studies, used in 17 studies, among which PASAT and SDMT were the most frequent cognition tests.

### PSQI and PASAT

PASAT is a valid cognitive assessment in MS which assessed auditory processing speed and working memory [85]. Seven studies have investigated the associations between the PASAT and PSQI. Aristotelous et al.’s linear regression showed that the PASAT score had an association with the performance score in PSQI [50]. Berard et al.’s results showed that sleep quality could be a predictor factor of decreased cognitive performance with sustained cognitive effort, which is defined as cognitive fatigue based on PASAT [52]. Ozkul et al. enrolled 112 MS in their study, and they couldn’t find any relationships between PASAT and PSQI [70]. According to Hughes et al.’s study in which 121 patients participated, the total PSQI was not associated with the PASAT score [61]. Fifty-three MS patients were enrolled in Mackay et al.’s study, and the findings showed that there was no statistically significant relation between PASAT and PSQI scores [66]. Siengsukon et al.’s results demonstrated that there was no association between cognitive fatigue through the PASAT test and sleep quality [76]. Evaluating the sleep quality in Whibley et al.’s study indicated that no associations were identified between PASAT scores and sleep domains [81]. Current evidence did not support a considerable link between sleep quality assessed by PSQI and auditory processing speed and working memory, based on PASAT in MS.

### PSQI and SDMT

SDMT assessed visual processing speed and working memory [85]. Evaluating the relation between SDMT and PSQI was accomplished in four studies. In Mackay et al.’s study, the results demonstrated no statistically significant relation between SDMT and sleep quality [66]. Ozkul et al.’s findings showed that there is no statistically significant relation between PSQI and SDMT scores in MS patients [70]. Siengsukon et al. also identified that patients with good sleep quality performed slightly better, but not statistically, on SDMT. Similarly, in Whibley et al.’s study, there weren’t any statistically significant associations between sleep variables and SDMT [76, 81]. It can be concluded that, based on the existing evidence, sleep quality cannot be considered a predictor of impaired visual processing speed and working memory, as evaluated by SDMT.

### PSQI and other cognitive assessments

Aldughmi et al. enrolled 51 MS patients and used the cognitive fatigability (based on continuous performance test, response speed variability [CPT-RSV]) test to demonstrate that cognitive performance was associated with only the daytime dysfunction component of the PSQI [49, 55]. Lamis et al. evaluated 77 MS patients’ cognitive function through the PDQ test, and the results showed that cognitive deficits, assessed by PDQ are associated with poorer sleep quality [63]. Laslett et al. enrolled 1717 patients and used MSSymS to evaluate their cognitive function, and they pointed out that the “fatigue and cognition” cluster is associated with poorer sleep quality [64]. According to Lehmann et al.’s study, in which 42 MS patients were included, n-back-hits and n-back-RT tests were used to evaluate the cognitive function, and the association between sleep disturbances and cognitive decline during tasks of sustained attention was observed [65]. Odintsova and Kopchak didn’t find a correlation in their study based on MoCA [68]. Riccitelli et al. enrolled 80 MS patients and used the BRB-N test to evaluate their cognitive function. They concluded that worse performances in the global, memory, and attention cognitive domains are correlated with poor sleep quality [72]. Sharhbanian et al. exhibited that cognitive deficits are significantly correlated with sleep disorders [75]. According to Siengsukon, Aldughmi, et al.’s results, visuospatial memory was correlated with poor sleep quality [76]. Siengsukon, Alshehri, et al.’s study revealed that bad sleepers have a significantly higher level of cognitive fatigue [77].

### ESS and cognitive outcomes

ESS is a simple, self-administered questionnaire that considers the patients’ general level of daytime sleepiness [86]. 

### ESS and SDMT

Braley et al. evaluated the cognitive function of 38 MS patients and the results indicated no association between ESS and SDMT [53]. Patel et al. evaluated the sleep quality and cognitive function in 102 MS patients and found that there are no differences in cognitive performance between MS participants with normal versus excessive daytime sleepiness [71]. The noted studies were the only evidence that assessed the possible association between daytime sleepiness assessed by ESS and visual processing speed and working memory, as evaluated by SDMT, which did not support a substantial affinity.

### ESS and other cognitive assessments

Laslett et al. enrolled 1717 MS patients, used the MSSymS test to assess cognitive function, and concluded that fatigue and cognition are associated with sleep quality [64]. In Sater et al.’s study, in which 32 MS patients’ cognitive function was evaluated with MFIS-Cognitive and NeuroTrax tests, ESS was mildly correlated with the cognitive component of the MFIS [73]. Siengsukon, Alshehri, et al. demonstrated that bad sleepers have a significantly higher level of cognitive fatigue [77].

### Objective sleep measures and cognitive outcomes

PSG is a multi-parameter kind of sleep investigation, which is considered the gold standard for diagnosing sleep-related breathing disorders [87]. Braley et al. in 2016 concluded that OSA and sleep disturbance are associated with memory, executive function, and processing speed [53]. In an assessment of 113 patients, Chinnadurai et al.‘s results showed a significant relationship between sleep impairments and cognitive fatigue [58]. Riccitelli et al. concluded that reduced REM sleep may affect attention abilities [72]. The findings of Sater et al.’s study also revealed that poor sleep efficacy may contribute to reduced cognitive function [73].

Actigraphy is also a valid and reliable instrument to assess sleep objectively, with approximately 90% agreement with polysomnography [88]. In three studies, actigraphy was used for sleep assessment. Aldughmi et al. showed that cognitive fatigability assessed by RSV is significantly associated with sleep efficiency and wake after sleep onset measures of actigraphy which is suggested to be mediated by depression [49]. The findings of Opelt et al.’s study revealed that discrepancies between self-report and actigraphy-based measures of sleep outcomes are linked with cognitive impairment in MS [69]. Whibley et al.’s outcomes pointed out associations between visual-spatial function and sleep duration and continuity [81].

Contrary to the self-reported techniques, it seems objective measurement of sleep condition, can significantly affect MS patients’ cognitive performance. By contrast, a polysomnographic study conducted by Anne-Laure Dubessy et al., found no significant association between central hyposmia and cognitive outcomes in MS. Cognitive evaluations using a poor-sensitive and non-specific method for MS, Mattis Dementia Rating Scale, and lack of appropriate addressing the possible confounders, may be the reasons for lack of significant findings in this study [59].

## Discussion

The objective of this systematic review was to provide an overview of the studies evaluating the relationship between sleep outcomes and cognition function in adults with MS, as assessed by either questionnaires or neuropsychological tests. Collectively, 35 studies were included in the final evaluation, and the majority of the studies discovered a link between poorer sleep quality and impairment in at least one domain of cognition in MS, which was more evident in the studies with objective measurement of sleep condition.

Despite the majority of evidence revealed a significant association between sleep conditions and cognitive abilities, a lack of a significant relationship was evident in some studies. The study conducted by Odintsova et al. used MoCA which is considered a sensitive and specific method for cognitive evolution in MS [68, 89], but there was a lack of appropriate addressing of the confounders in this study. Ozkul et al.’s research only reported the correlations between cognitive scales and PSQI as one of the secondary outcomes of the study with no control for cofounders [70]. Chen et al.‘s study also investigated the short-term association between prior night sleep measures and cognitive outcomes and could not detect a considerable link in this regard [90]. Therefore, this contrast can be explained by confounding factors influencing the relationship between sleep and cognition as well as using the different cognitive and sleep assessment tools with a wide range of specificity or sensitivity, and the difference between subjective and objective evaluations. Despite the observed associations between sleep quality, assessed by PSQI and cognitive outcomes, current evidence did not support a substantial association between sleep quality assessed by PSQI and processing speed and working memory in patients with MS.

RLS is found to be associated with an increased risk of incidence of all-cause dementia in older adults [91]. RLS is frequent among patients with MS [24, 92]. Cederberg et al. in 2020 enrolled 275 MS patients and used the MSNQ test to evaluate the cognitive function, and demonstrated that sleep disturbance may be an intermediary factor in the connection between RLS and cognitive impairment, and in 2022 concluded that those with more severe RLS may experience worse cognitive function, particularly slower processing speed and more memory difficulties [55, 56, 93].

Evidence regarding the association between insomnia and the risk of cognitive dysfunction in the general population is not deterministic yet [90, 94]. Regarding MS, Hare et al. revealed a significant relationship between insomnia and cognitive function, which is only mediated by fatigue catastrophizing [91]. Schellaert et al.‘s findings showed that cognitive factors were associated with insomnia disorder [92]. Sumowski et al.‘s study also connected sleep disturbance to poor memory [93]. Future studies are needed to discover the possible link between insomnia and different aspects of cognitive function in MS.

Recent studies have reported that sleep disruption deteriorates cognitive performance by facilitating the pathogenesis of AD throughout the entire course of AD, from the preclinical to the advanced phase [95]. It is widely known that MCI and developing AD are both linked with sleep and circadian rhythm disturbances, making the management of sleep disturbance crucial [96]. Memory redistribution interruption while sleeping, is suggested as the consequence of prefrontal dysfunction observed in dementia and cognitive impairment conditions [97]. Also, recent evidence suggests that sleep disruption is correlated with disturbed verbal fluency, executive function, attention, spatial memory, and processing speed, particularly in AD patients [98–101]. A recent systematic review concluded that REM-sleep decline status is associated with Alzheimer’s Disease (AD) cognitive impairments [102]. Such an association was also evident in MS patients, too [58, 72].

Cognitive impairment is considered to be poorly managed in patients with MS [103]. A recent systematic literature review found minimal efficacy of pharmacological interventions in the management of MS-related cognitive dysfunction [104]. Despite the spectacular progress in MS management, current strategies confer only partial protection against the neurodegenerative component of MS [14], which is significantly correlated to sleep condition [105]. The observed association, suggests sleep is a modifiable risk factor for cognitive dysfunction which can be targeted to improve neuropsychiatric outcomes in MS [106]. A longitudinal study conducted by McNicholas et al. demonstrated the potential for OSA treatment to improve verbal learning in people with MS [67]. In addition, cognitive efficacy of the interventions for improving the sleep condition such as melatonin supplementation was suggested [107–111]; however, there is a lack of evidence regarding the effect of treatment methods for sleep management in MS which shed light on the importance of future studies on this topic [112]. In addition, improving sleep status is challenges in MS. For example, physical activity has been discussed as an approach to expanding sleep quality which is not sufficient for the MS population [93]. Through understanding the cause of sleep disorders and their consequences, specific meditations and treatment can be obtained. Anxiety, depression, and neuropathic pain are among the initial symptoms concerning sleep quality [113], which can lead to cognitive dysfunction.

Given the limitations of the included studies, findings of this study need to be interpreted with caution. The majority of them were cross-sectional studies, which provided no insight into when exactly the association between sleep and cognition in MS patients first appeared, whether it was immediately following diagnosis or during treatment courses. Additionally, most studies did not provide clear information on confounding variables, medical history, comorbidities, and medication use, all of which could have influenced the relationship between sleep and cognition. To address this issue, empirical validations are necessary to establish whether successful interventions for improving sleep quality are aimed at enhancing cognitive functioning and confirm the link between sleep disturbance and cognitive dysfunction.

## Data Availability

All data generated or analyzed during this study are included in this published article.

## References

[1] McGinley MP, Goldschmidt CH, Rae-Grant AD. Diagnosis and treatment of multiple sclerosis: a review. JAMA. 2021;325(8):765–79. 10.1001/jama.2020.26858. 33620411 10.1001/jama.2020.26858

[2] Walton C, King R, Rechtman L, Kaye W, Leray E, Marrie RA, et al. Rising prevalence of multiple sclerosis worldwide: insights from the Atlas of MS, third edition. Mult Scler. 2020;26(14):1816–21. 10.1177/1352458520970841. 33174475 10.1177/1352458520970841 PMC7720355

[3] Thompson AJ, Banwell BL, Barkhof F, Carroll WM, Coetzee T, Comi G, et al. Diagnosis of multiple sclerosis: 2017 revisions of the McDonald criteria. Lancet Neurol. 2018;17(2):162–73. 10.1016/S1474-4422(17)30470-2. 29275977 10.1016/S1474-4422(17)30470-2

[4] Carroll WM. 2017 McDonald MS diagnostic criteria: evidence-based revisions. Mult Scler J. 2018;24(2):92–5. 10.1177/1352458517751861. 10.1177/1352458517751861 29451442

[5] Tahmasbi F, Salehi-Pourmehr H, Hajebrahimi S, Soleimanzadeh F. Effects of tibial nerve electrical stimulation on bowel dysfunction in multiple sclerosis: a systematic review. Int J Drug Res Clin. 2023;1(1):e24 e. 10.34172/ijdrc.2023.e24.

[6] Tahmasbi F, Hosseini S, Hajebrahimi S, Heris RM, Salehi-Pourmehr H. Efficacy of tibial nerve stimulation in neurogenic lower urinary tract dysfunction among patients with multiple sclerosis: a systematic review and meta-analysis. Urol Res Pract. 2023;49(2):100–11. 10.5152/tud.2023.22241. 37877856 10.5152/tud.2023.22241 PMC10192727

[7] Chisari CG, Sgarlata E, Arena S, D’Amico E, Toscano S, Patti F. An update on the pharmacological management of pain in patients with multiple sclerosis. Expert Opin Pharmacother. 2020;21(18):2249–63. 10.1080/14656566.2020.1757649. 32343626 10.1080/14656566.2020.1757649

[8] Abbasi H, Shakouri F, Mosaddeghi-Heris R, Gholipour-Khalili E, Jahanshahlou F, Sanaie S, et al. Mediterranean-like diets in multiple sclerosis: a systematic review. Rev Neurol. 2023. 10.1016/j.neurol.2023.07.017. 10.1016/j.neurol.2023.07.017 39492055

[9] Langer-Gould AM, Smith JB, Gonzales EG, Piehl F, Li BH. Multiple sclerosis, disease-modifying therapies, and infections. Neurol Neuroimmunol Neuroinflammation. 2023;10(6): e200164. 10.1212/NXI.0000000000200164. 10.1212/NXI.0000000000200164 PMC10574822 37813594

[10] Wu X, Wang S, Xue T, Tan X, Li J, Chen Z, et al. Disease-modifying therapy in progressive multiple sclerosis: a systematic review and network meta-analysis of randomized controlled trials. Front Neurol. 2024;15(1295770): 1295770. 10.3389/fneur.2024.1295770. 38529035 10.3389/fneur.2024.1295770 PMC10962394

[11] Drudge C, Samjoo IA, Brennan R, Badgujar L, Khurana V, Tiwari S, et al. An overview of reviews with network meta-analyses comparing disease-modifying therapies for relapsing multiple sclerosis. Future Neurol. 2023;18(3):FNL70. 10.2217/fnl-2023-0009.

[12] Feige J, Moser T, Bieler L, Schwenker K, Hauer L, Sellner J. Vitamin D supplementation in multiple sclerosis: a critical analysis of potentials and threats. Nutrients. 2020;12(3): 783. 10.3390/nu12030783. 32188044 10.3390/nu12030783 PMC7146466

[13] Tryfonos C, Mantzorou M, Fotiou D, Vrizas M, Vadikolias K, Pavlidou E, et al. Dietary supplements on controlling multiple sclerosis symptoms and relapses: current clinical evidence and future perspectives. Med (Basel). 2019;6(3). 10.3390/medicines6030095. 10.3390/medicines6030095 PMC6789617 31547410

[14] Hauser SL, Cree BAC. Treatment of multiple sclerosis: a review. Am J Med. 2020;133(12):1380-90.e2. 10.1016/j.amjmed.2020.05.049. 32682869 10.1016/j.amjmed.2020.05.049 PMC7704606

[15] Lunde HMB, Assmus J, Myhr KM, Bø L, Grytten N. Survival and cause of death in multiple sclerosis: a 60-year longitudinal population study. J Neurol Neurosurg Psychiatry. 2017;88(8):621–5. 10.1136/jnnp-2016-315238. 28365589 10.1136/jnnp-2016-315238 PMC5537547

[16] Veauthier C. Sleep disorders in multiple sclerosis. Review. Curr Neurol Neurosci Rep. 2015;15(5):21. 10.1007/s11910-015-0546-0. 25773000 10.1007/s11910-015-0546-0

[17] Åkerstedt T, Olsson T, Alfredsson L, Hedström AK. Insufficient sleep during adolescence and risk of multiple sclerosis: results from a Swedish case-control study. J Neurol Neurosurg Psychiatry. 2023;94(5):331–6. 10.1136/jnnp-2022-330123. 36690431 10.1136/jnnp-2022-330123 PMC10176406

[18] Yazdchi M, Khanalizadeh R, Nasiri E, Naseri A, Talebi M, Talebi M. Sleep status in multiple sclerosis: role of vitamin D and body mass index. Curr J Neurol. 2022;21(2):66–73. 10.18502/cjn.v21i2.10489. 38011482 10.18502/cjn.v21i2.10489 PMC9860209

[19] Zhang GX, Zhang WT, Gao SS, Zhao RZ, Yu WJ, Izquierdo G. Sleep disorders in patients with multiple sclerosis in Spain. Neurología. 2024;39(1):29–35. 10.1016/j.nrl.2021.03.012. 38161070 10.1016/j.nrleng.2021.03.011

[20] Moradi A, Ebrahimian A, Naseri A, Talebi M. Multiple sclerosis patients has lower sleep quality; a systematic review and meta-analysis. J Neurol Sci. 2023;455:101016jjns2023121871.

[21] Zeng X, Dorstyn DS, Edwards G, Kneebone I. The prevalence of insomnia in multiple sclerosis: a meta-analysis. Sleep Med Rev. 2023;72: 101842. 10.1016/j.smrv.2023.101842. 37660580 10.1016/j.smrv.2023.101842

[22] Čarnická Z, Kollár B, Šiarnik P, Krížová L, Klobučníková K, Turčáni P. Sleep disorders in patients with multiple sclerosis. J Clin Sleep Med. 2015;11(5):553–7. 10.5664/jcsm.4702. 25700869 10.5664/jcsm.4702 PMC4410929

[23] Zhang Y, Ren R, Yang L, Zhang H, Shi Y, Vitiello MV, et al. Sleep in multiple sclerosis: a systematic review and meta-analysis of polysomnographic findings. J Clin Sleep Med. 2023;19(2):253–65. 10.5664/jcsm.10304. 36117421 10.5664/jcsm.10304 PMC9892728

[24] Mnif S, Saoussen D, Nadia B, Nouha F, Sonda MK, Salma S, et al. Sleep disorders in relapsing-remitting multiple sclerosis patients. Mult Scler Relat Disorders. 2023;71: 104334. 10.1016/j.msard.2022.104334.

[25] Sumowski JF, Benedict R, Enzinger C, Filippi M, Geurts JJ, Hamalainen P, et al. Cognition in multiple sclerosis: state of the field and priorities for the future. Neurology. 2018;90(6):278–88. 10.1212/wnl.0000000000004977. 29343470 10.1212/WNL.0000000000004977 PMC5818015

[26] Portaccio E, Amato MP. Cognitive impairment in multiple sclerosis: an update on assessment and management. NeuroSci. 2022;3(4):667–76. 10.3390/neurosci3040048. 10.3390/neurosci3040048 PMC11523737 39483763

[27] Talebi M, Sadigh-Eteghad S, Talebi M, Naseri A, Zafarani F. Predominant domains and associated demographic and clinical characteristics in multiple sclerosis-related cognitive impairment in mildly disabled patients. Egypt J Neurol Psychiatry Neurosurg. 2022;58(1):48. 10.1186/s41983-022-00485-7.

[28] Podda J, Ponzio M, Pedullà L, Monti Bragadin M, Battaglia MA, Zaratin P, et al. Predominant cognitive phenotypes in multiple sclerosis: insights from patient-centered outcomes. Multiple Scler Relat Disorders. 2021;51:102919. 10.1016/j.msard.2021.102919. 10.1016/j.msard.2021.102919 33799285

[29] Wu W, Francis H, Lucien A, Wheeler T-A, Gandy M. The prevalence of cognitive impairment in relapsing-remitting multiple sclerosis: a systematic review and meta-analysis. Neuropsychol Rev. 2024. 10.1007/s11065-024-09640-8. 38587704 10.1007/s11065-024-09640-8 PMC12328523

[30] Dastgheib M, Kulanayagam A, Dringenberg HC. Is the role of sleep in memory consolidation overrated? Neurosci Biobehav Rev. 2022;140: 104799. 10.1016/j.neubiorev.2022.104799. 35905801 10.1016/j.neubiorev.2022.104799

[31] Belia M, Keren-Portnoy T, Vihman M. Systematic review of the effects of sleep on memory and word learning in infancy. Lang Learn. 2023;73(2):613–51. 10.1111/lang.12544.

[32] Newbury CR, Crowley R, Rastle K, Tamminen J. Sleep deprivation and memory: meta-analytic reviews of studies on sleep deprivation before and after learning. Psychol Bull. 2021;147(11):1215–40. 10.1037/bul0000348. 35238586 10.1037/bul0000348 PMC8893218

[33] Helfer B, Bozhilova N, Cooper RE, Douzenis JI, Maltezos S, Asherson P. The key role of daytime sleepiness in cognitive functioning of adults with attention deficit hyperactivity disorder. Eur Psychiatry. 2020;63(1): e31. 10.1192/j.eurpsy.2020.28. 32131909 10.1192/j.eurpsy.2020.28 PMC7315868

[34] Rosenberg R, Thorpy MJ, Doghramji K, Morse AM. Brain fog in central disorders of hypersomnolence: a review. J Clin Sleep Med. 2024;20(4):643–51. 10.5664/jcsm.11014. 38217475 10.5664/jcsm.11014 PMC10985301

[35] Hughes AJ, Dunn KM, Chaffee T. Sleep disturbance and cognitive dysfunction in multiple sclerosis: a systematic review. Curr Neurol Neurosci Rep. 2018;18(1):2. 10.1007/s11910-018-0809-7. 29380072 10.1007/s11910-018-0809-7

[36] Aromataris E MZE. JBI manual for evidence synthesis. 2020. Available from 10.46658/JBIMES-20-01.

[37] Page MJ, McKenzie JE, Bossuyt PM, Boutron I, Hoffmann TC, Mulrow CD, et al. The PRISMA 2020 statement: an updated guideline for reporting systematic reviews. BMJ. 2021;372(71):10.1136/bmj.n71. 10.1136/bmj.n71 PMC8005924 33782057

[38] Ouzzani M, Hammady H, Fedorowicz Z, Elmagarmid A. Rayyan—a web and mobile app for systematic reviews. Systematic Reviews. 2016;5:5;10.1186/s13643-016-0384–4. 27919275 10.1186/s13643-016-0384-4 PMC5139140

[39] Moola S, Munn Z, Tufanaru C, Aromataris E, Sears K, Sfetcu R, Currie M, Lisy K, Qureshi R, Mattis P, Mu P. Systematic reviews of etiology and risk (2020). Aromataris E, Lockwood C, Porritt K, Pilla B, Jordan Z, editors. JBI Manual for Evidence Synthesis. JBI; 2024. Available from: https://synthesismanual.jbi.global. 10.46658/JBIMES-24-06.

[40] Bahmani DS, Kesselring J, Papadimitriou M, Bansi J, Pühse U, Gerber M, et al. In patients with multiple sclerosis, both objective and subjective sleep, depression, fatigue, and paresthesia improved after 3 weeks of regular exercise. Front Psychiatry. 2019;10(MAY):265. 10.3389/fpsyt.2019.00265. 31130879 10.3389/fpsyt.2019.00265 PMC6510171

[41] Hu M, Muhlert N, Robertson N, Winter M. Perceived fatigue and cognitive performance change in multiple sclerosis: uncovering predictors beyond baseline fatigue. Mult Scler Relat Disord. 2019;32:46–53. 10.1016/j.msard.2019.04.011. 31030019 10.1016/j.msard.2019.04.011

[42] Bruce JM, Arnett P. Clinical correlates of generalized worry in multiple sclerosis. J Clin Exp Neuropsychol. 2009;31(6):698–705. 10.1080/13803390802484789. 19107677 10.1080/13803390802484789

[43] Cehelyk EK, Harvey DY, Grubb ML, Jalel R, El-Sibai MS, Markowitz CE, et al. Uncovering the association between fatigue and fatigability in multiple sclerosis using cognitive control. Mult Scler Relat Disord. 2019;27:269–75. 10.1016/j.msard.2018.10.112. 30423531 10.1016/j.msard.2018.10.112 PMC6442685

[44] Jain V, Arunkumar A, Kingdon C, Lacerda E, Nacul L. Prevalence of and risk factors for severe cognitive and sleep symptoms in ME/CFS and MS. BMC Neurol. 2017;17(1):117. 10.1186/s12883-017-0896-0. 28633629 10.1186/s12883-017-0896-0 PMC5477754

[45] Turkoglu R, Benbir G, Ozyurt S, Arsoy E, Akbayir E, Turan S, et al. Sleep disturbance and cognitive decline in multiple sclerosis patients with isolated optic neuritis as the first demyelinating event. International Ophthalmology. 2020;40(1):151–8. 10.1007/s10792-019-01157-x. 31432354 10.1007/s10792-019-01157-x

[46] Hoogerwerf AEW, Bol Y, Lobbestael J, Hupperts R, van Heugten CM. Mindfulness-based cognitive therapy for severely fatigued multiple sclerosis patients: a waiting list controlled study. J Rehabil Med. 2017;49(6):497–504. 10.2340/16501977-2237. 28597907 10.2340/16501977-2237

[47] Bahmani DS, Esmaeili L, Shaygannejad V, Gerber M, Kesselring J, Lang UE, et al. Stability of mental toughness, sleep disturbances, and physical activity in patients with multiple sclerosis (MS)-A longitudinal and pilot study. Front Psychiatry. 2018;9(MAY):182. 10.3389/fpsyt.2018.00182. 29867606 10.3389/fpsyt.2018.00182 PMC5966704

[48] Arnett PA. Longitudinal consistency of the relationship between depression symptoms and cognitive functioning in multiple sclerosis. CNS Spectr. 2005;10(5):372 – 82;10.1017/S1092852900022744. 15858455 10.1017/s1092852900022744

[49] Aldughmi M, Huisinga J, Lynch SG, Siengsukon CF. The relationship between fatigability and sleep quality in people with multiple sclerosis. Mult Scler J Exp Transl Clin. 2016;2(2055217316682774):10.1177/2055217316682774. 10.1177/2055217316682774 PMC5433332 28607747

[50] Aristotelous P, Stefanakis M, Pantzaris M, Pattichis C, Hadjigeorgiou GM, Giannaki CD. Associations between functional capacity, isokinetic leg strength, sleep quality and cognitive function in multiple sclerosis patients: a cross-sectional study. Postgrad Med. 2019;131(7):453–60. 10.1080/00325481.2019.1662271. 31469966 10.1080/00325481.2019.1662271

[51] Beier M, Amtmann D, Ehde DM. Beyond depression: predictors of self-reported cognitive function in adults living with MS. Rehabil Psychol. 2015;60(3):254. 26192051 10.1037/rep0000045 PMC4564347

[52] Berard JA, Smith AM, Walker LAS. Predictive models of cognitive fatigue in multiple sclerosis. Arch Clin Neuropsychol. 2019;34(1):31–8. 10.1093/arclin/acy014. 29471423 10.1093/arclin/acy014

[53] Braley TJ, Kratz AL, Kaplish N, Chervin RD. Sleep and cognitive function in multiple sclerosis. Sleep. 2016;39(8):1525–33. 10.5665/sleep.6012. 27166237 10.5665/sleep.6012 PMC4945311

[54] Braley TJ, Shieu MM, Zaheed AB, Dunietz GL. Pathways between multiple sclerosis, sleep disorders, and cognitive function: Longitudinal findings from The Nurses’ Health Study. Mult Scler. 2023;29(3):436–46. 10.1177/13524585221144215. 36633265 10.1177/13524585221144215 PMC9991978

[55] Cederberg KLJ, Jeng B, Sasaki JE, Motl RW. Restless legs syndrome, sleep quality, and perceived cognitive impairment in adults with multiple sclerosis. Mult Scler Relat Disord. 2020;43(102176):10.1016/j.msard.2020.102176. 10.1016/j.msard.2020.102176 PMC7363523 32498034

[56] Cederberg KLJ, Mathison B, Schuetz ML, Motl RW. Restless legs syndrome severity and cognitive function in adults with multiple sclerosis: an exploratory pilot study. Int J MS Care. 2022;24(4):154–61. 10.7224/1537-2073.2020-120. 35875462 10.7224/1537-2073.2020-120 PMC9296052

[57] Chen MH, Cherian C, Elenjickal K, Rafizadeh CM, Ross MK, Leow A, et al. Real-time associations among MS symptoms and cognitive dysfunction using ecological momentary assessment. Front Med (Lausanne). 2022;9(1049686):10.3389/fmed.2022.1049686. 10.3389/fmed.2022.1049686 PMC9877417 36714150

[58] Chinnadurai SA, Gandhirajan D, Pamidimukala V, Kesavamurthy B, Venkatesan SA. Analysing the relationship between polysomnographic measures of sleep with measures of physical and cognitive fatigue in people with multiple sclerosis. Mult Scler Relat Disord. 2018;24:32–7. 10.1016/j.msard.2018.05.016. 29883851 10.1016/j.msard.2018.05.016

[59] Dubessy AL, du Montcel ST, Viala F, Assouad R, Tiberge M, Papeix C, et al. Association of central hypersomnia and fatigue in patients with multiple sclerosis a polysomnographic study. Neurology. 2021;97(1):E23–33. 10.1212/wnl.0000000000012120. 33931534 10.1212/WNL.0000000000012120

[60] Hare CJ, Crangle CJ, Carney CE, Hart T. Insomnia symptoms. Subjective appraisals, and fatigue: a multiple mediation model. Behav Sleep Med. 2019;17(3):269–80. 10.1080/15402002.2017.1342167. 28609122 10.1080/15402002.2017.1342167

[61] Hughes AJ, Parmenter BA, Haselkorn JK, Lovera JF, Bourdette D, Boudreau E, et al. Sleep and its associations with perceived and objective cognitive impairment in individuals with multiple sclerosis. J Sleep Res. 2017;26(4):428–35. 10.1111/jsr.12490. 28093823 10.1111/jsr.12490

[62] Hughes AJ, Turner AP, Alschuler KN, Atkins DC, Beier M, Amtmann D, et al. Association between sleep problems and perceived cognitive dysfunction over 12 months in individuals with multiple sclerosis. Behav Sleep Med. 2018;16(1):79–91. 10.1080/15402002.2016.1173553. 27167969 10.1080/15402002.2016.1173553

[63] Lamis DA, Hirsch JK, Pugh KC, Topciu R, Nsamenang SA, Goodman A, et al. Perceived cognitive deficits and depressive symptoms in patients with multiple sclerosis: perceived stress and sleep quality as mediators. Mult Scler Relat Disord. 2018;25:150–5. 10.1016/j.msard.2018.07.019. 30081314 10.1016/j.msard.2018.07.019

[64] Laslett LL, Honan C, Turner JA, Dagnew B, Campbell JA, Gill TK et al. Poor sleep and multiple sclerosis: associations with symptoms of multiple sclerosis and quality of life. J Neurol Neurosurg Psychiatry. 2022. 10.1136/jnnp-2022-329227. 10.1136/jnnp-2022-329227 35896381

[65] Lehmann P, Eling P, Kastrup A, Grothues O, Hildebrandt H. Self-reported sleep problems, but not fatigue, lead to decline in sustained attention in MS patients. Mult Scler. 2013;19(4):490–7. 10.1177/1352458512457719. 22933623 10.1177/1352458512457719

[66] Mackay L, Johnson AM, Moodie ST, Rosehart H, Morrow SA. Predictors of cognitive fatigue and fatigability in multiple sclerosis. Mult Scler Relat Disord. 2021;56:103316. 10.1016/j.msard.2021.103316. 34638096 10.1016/j.msard.2021.103316

[67] McNicholas N, Russell A, Nolan G, Tubridy N, Hutchinson M, Garvey JF, et al. Impact of obstructive sleep apnoea on cognitive function in multiple sclerosis: a longitudinal study. J Sleep Res. 2021;30(3): e13159. 10.1111/jsr.13159. 32791570 10.1111/jsr.13159

[68] Odintsova TA, Kopchak OO. Sleep disorders in relapsing-remitting multiple sclerosis patients. Wiad Lek. 2021;74(2):257–6233813482. 33813482

[69] Opelt BL, Lewis C, Hughes AJ. Discrepancies between self-report and objective sleep outcomes are associated with cognitive impairment and fatigue in people with multiple sclerosis and insomnia. Mult Scler Relat Disord. 2023;71(104588):10.1016/j.msard.2023.104588. 10.1016/j.msard.2023.104588 36841176

[70] Ozkul C, Guclu-Gunduz A, Eldemir K, Apaydin Y, Yazici G, Irkec C. Clinical features and physical performance in multiple sclerosis patients with and without cognitive impairment: a cross-sectional study. Int J Rehabil Res. 2020;43(4):316–23. 10.1097/mrr.0000000000000428. 32804701 10.1097/MRR.0000000000000428

[71] Patel VP, Walker LA, Feinstein A. Processing speed and distractibility in multiple sclerosis: the role of sleep. Mult Scler Relat Disord. 2017;11:40–2. 10.1016/j.msard.2016.11.012. 28104254 10.1016/j.msard.2016.11.012

[72] Riccitelli GC, Pacifico D, Manconi M, Sparasci D, Sacco R, Gobbi C, et al. Relationship between cognitive disturbances and sleep disorders in multiple sclerosis is modulated by psychiatric symptoms. Mult Scler Relat Disord. 2022;64(103936):10.1016/j.msard.2022.103936. 10.1016/j.msard.2022.103936 35717899

[73] Sater RA, Gudesblatt M, Kresa-Reahl K, Brandes DW, Sater PA. The relationship between objective parameters of sleep and measures of fatigue, depression, and cognition in multiple sclerosis. Mult Scler J Exp Transl Clin. 2015;1(2055217315577828):10.1177/2055217315577828. 10.1177/2055217315577828 PMC5433423 28607689

[74] Schellaert V, Labauge P, Lebrun C, Maudarbocus KH, Bernard J, Blache JB et al. Psychological processes associated with insomnia in patients with multiple sclerosis. Sleep. 2018;41(3). 10.1093/sleep/zsy002 10.1093/sleep/zsy002 29309702

[75] Shahrbanian S, Duquette P, Kuspinar A, Mayo NE. Contribution of symptom clusters to multiple sclerosis consequences. Qual Life Res. 2015;24(3):617–29. 10.1007/s11136-014-0804-7. 25228080 10.1007/s11136-014-0804-7

[76] Siengsukon CF, Aldughmi M, Kahya M, Lynch S, Bruce J, Glusman M, et al. Individuals with mild MS with poor sleep quality have impaired visuospatial memory and lower perceived functional abilities. Disabil Health J. 2018;11(1):116–21. 10.1016/j.dhjo.2017.04.011. 28495217 10.1016/j.dhjo.2017.04.011

[77] Siengsukon CF, Alshehri M, Aldughmi M. Self-report sleep quality combined with sleep time variability distinguishes differences in fatigue, anxiety, and depression in individuals with multiple sclerosis: a secondary analysis. Mult Scler J Exp Transl Clin. 2018;4(4):2055217318815924. 10.1177/2055217318815924. 30559974 10.1177/2055217318815924 PMC6293381

[78] Sumowski JF, Horng S, Brandstadter R, Krieger S, Leavitt VM, Sand IK, et al. Sleep disturbance and memory dysfunction in early multiple sclerosis. Annals of Clinical and Translational Neurology. 2021;8(6):1172–82. 10.1002/acn3.51262. 33951348 10.1002/acn3.51262 PMC8164863

[79] van Geest Q, Westerik B, van der Werf YD, Geurts JJ, Hulst HE. The role of sleep on cognition and functional connectivity in patients with multiple sclerosis. J Neurol. 2017;264(1):72–80. 10.1007/s00415-016-8318-6. 27778159 10.1007/s00415-016-8318-6 PMC5225184

[80] Valentine TR, Kratz AL, Kaplish N, Chervin RD, Braley TJ. Sleep-disordered breathing and neurocognitive function in multiple sclerosis: differential associations across cognitive domains. Mult Scler. 2023;29(7):832 – 45;10.1177/13524585231169465. 37194432 10.1177/13524585231169465

[81] Whibley D, Goldstein C, Kratz AL, Braley TJ. A multidimensional approach to sleep health in multiple sclerosis. Mult Scler Relat Disord. 2021;56(103271):10.1016/j.msard.2021.103271. 10.1016/j.msard.2021.103271 PMC8678321 34614459

[82] Borragán G, Gilson M, Atas A, Slama H, Lysandropoulos A, De Schepper M, et al. Cognitive fatigue, sleep and cortical activity in multiple sclerosis disease. a behavioral, polysomnographic and functional near-infrared spectroscopy investigation. Front Hum Neurosci. 2018;12(378):10.3389/fnhum.2018.00378. 10.3389/fnhum.2018.00378 PMC6158319 30294266

[83] Terauchi T, Mizuno M, Suzuki M, Akasaka H, Maeta M, Tamura K, Hosokawa K, Nishijima T, Maeda T. Clinical features of sleep apnea syndrome and cognitive impairment in multiple sclerosis. Mult Scler Relat Disord. 2024;82:105407. 10.1016/j.msard.2023.105407. Epub 2023 Dec 23. 10.1016/j.msard.2023.105407 38160637

[84] Buysse DJ, Reynolds CF 3, Monk TH, Berman SR, Kupfer DJ. The Pittsburgh Sleep Quality Index: a new instrument for psychiatric practice and research. Psychiatry Res. 1989;28(2):193–213 10.1016/0165–1781(89)90047-4. 2748771 10.1016/0165-1781(89)90047-4

[85] Naseri A, Forghani N, Sadigh-Eteghad S, Shanehbandi D, Asadi M, Nasiri E, et al. Circulatory antioxidant and oxidative stress markers are in correlation with demographics but not cognitive functions in multiple sclerosis patients. Mult Scler Relat Disord. 2022;57(103432):10.1016/j.msard.2021.103432. 10.1016/j.msard.2021.103432 34922253

[86] Johns MW. A new method for measuring daytime sleepiness: the Epworth sleepiness scale. Sleep. 1991;14(6):540–5. 10.1093/sleep/14.6.540. 1798888 10.1093/sleep/14.6.540

[87] Rundo JV, Downey R 3rd. Polysomnography. Handb Clin Neurol. 2019;160:381–92. 10.1016/b978-0-444-64032-1.00025-4. 31277862 10.1016/B978-0-444-64032-1.00025-4

[88] Berger AM, Wielgus KK, Young-McCaughan S, Fischer P, Farr L, Lee KA. Methodological challenges when using actigraphy in research. J Pain Symptom Manag. 2008;36(2):191–9. 10.1016/j.jpainsymman.2007.10.008. 10.1016/j.jpainsymman.2007.10.008 PMC2542506 18400460

[89] Rosca EC, Simu M. Montreal cognitive assessment for evaluating cognitive impairment in multiple sclerosis: a systematic review. Acta Neurol Belg. 2020;120(6):1307–21. 10.1007/s13760-020-01509-w. 32996098 10.1007/s13760-020-01509-w

[90] Zhao JL, Cross N, Yao CW, Carrier J, Postuma RB, Gosselin N, et al. Insomnia disorder increases the risk of subjective memory decline in middle-aged and older adults: a longitudinal analysis of the Canadian longitudinal study on aging. Sleep. 2022;45(11):zsac176. 10.1093/sleep/zsac176. 35877203 10.1093/sleep/zsac176 PMC9644124

[91] Kim KY, Kim EH, Lee M, Ha J, Jung I, Kim E. Restless leg syndrome and risk of all-cause dementia: a nationwide retrospective cohort study. Alzheimer’s Research and Therapy. 2023;15(1):46. 10.1186/s13195-023-01191-z. 10.1186/s13195-023-01191-z PMC9987068 36879327

[92] Aljarallah S, Alkhawajah N, Aldosari O, Alhuqbani M, Alqifari F, Alkhuwaitir B, et al. Restless leg syndrome in multiple sclerosis: a case-control study. Front Neurol. 2023;14(1194212):10.3389/fneur.2023.1194212. 10.3389/fneur.2023.1194212 PMC10315471 37404942

[93] Cederberg KLJ, Jeng B, Sasaki JE, Sikes EM, Cutter G, Motl RW. Physical activity and self-reported sleep quality in adults with multiple sclerosis. Disabil Health J. 2021;14(4): 101133. 10.1016/j.dhjo.2021.101133. 34193388 10.1016/j.dhjo.2021.101133 PMC8448914

[94] Selbaek-Tungevåg S, Selbaek G, Strand BH, Myrstad C, Livingston G, Lydersen S, et al. Insomnia and risk of dementia in a large population-based study with 11-year follow-up: the HUNT study. J Sleep Res. 2023;32(4): e13820. 10.1111/jsr.13820. 36689779 10.1111/jsr.13820

[95] Ju YE, Lucey BP, Holtzman DM. Sleep and Alzheimer disease pathology–a bidirectional relationship. Nat Rev Neurol. 2014;10(2):115–9. 10.1038/nrneurol.2013.269. 24366271 10.1038/nrneurol.2013.269 PMC3979317

[96] Kang DW, Lee CU, Lim HK. Role of sleep disturbance in the trajectory of Alzheimer’s disease. Clin Psychopharmacol Neurosci. 2017;15(2):89–99. 10.9758/cpn.2017.15.2.89. 28449556 10.9758/cpn.2017.15.2.89 PMC5426492

[97] da Silva RA. Sleep disturbances and mild cognitive impairment: a review. Sleep Sci. 2015;8(1):36–41. 10.1016/j.slsci.2015.02.001. 26483941 10.1016/j.slsci.2015.02.001 PMC4608881

[98] Zhu B, Dong Y, Xu Z, Gompf HS, Ward SA, Xue Z, et al. Sleep disturbance induces neuroinflammation and impairment of learning and memory. Neurobiol Dis. 2012;48(3):348–55. 10.1016/j.nbd.2012.06.022. 22776332 10.1016/j.nbd.2012.06.022 PMC3461115

[99] Lim AS, Kowgier M, Yu L, Buchman AS, Bennett DA. Sleep fragmentation and the risk of incident Alzheimer’s Disease and cognitive decline in older persons. Sleep. 2013;36(7):1027–32. 10.5665/sleep.2802. 23814339 10.5665/sleep.2802 PMC3669060

[100] Emamian F, Khazaie H, Tahmasian M, Leschziner GD, Morrell MJ, Hsiung GY, et al. The association between obstructive sleep apnea and Alzheimer’s disease: a meta-analysis perspective. Front Aging Neurosci. 2016;8(78):10.3389/fnagi.2016.00078. 10.3389/fnagi.2016.00078 PMC4828426 27148046

[101] Yaffe K, Laffan AM, Harrison SL, Redline S, Spira AP, Ensrud KE, et al. Sleep-disordered breathing, hypoxia, and risk of mild cognitive impairment and dementia in older women. Jama. 2011;306(6):613–9. 10.1001/jama.2011.1115. 21828324 10.1001/jama.2011.1115 PMC3600944

[102] Zhang Y, Ren R, Yang L, Zhang H, Shi Y, Okhravi HR, et al. Sleep in Alzheimer’s disease: a systematic review and meta-analysis of polysomnographic findings. Translational Psychiatry. 2022;12(1):136. 10.1038/s41398-022-01897-y. 35365609 10.1038/s41398-022-01897-y PMC8976015

[103] DeLuca J, Chiaravalloti ND, Sandroff BM. Treatment and management of cognitive dysfunction in patients with multiple sclerosis. Nature Reviews Neurology. 2020;16(6):319–32. 10.1038/s41582-020-0355-1. 32372033 10.1038/s41582-020-0355-1

[104] Motavalli A, Majdi A, Hosseini L, Talebi M, Mahmoudi J, Hosseini SH, et al. Pharmacotherapy in multiple sclerosis-induced cognitive impairment: a systematic review and meta-analysis. Multiple Scler Relat Disorders. 2020;46: 102478. 10.1016/j.msard.2020.102478. 10.1016/j.msard.2020.102478 32896820

[105] Ferini-Strambi L, Liguori C, Lucey BP, Mander BA, Spira AP, Videnovic A, et al. Role of sleep in neurodegeneration: the consensus report of the 5th Think Tank World Sleep Forum. Neurological Sciences. 2024;45(2):749–67. 10.1007/s10072-023-07232-7. 38087143 10.1007/s10072-023-07232-7

[106] Novak AM, Lev-Ari S. Resilience, stress, well-being, and sleep quality in multiple sclerosis. J Clin Med. 2023;12(2):716. 10.3390/jcm12020716. 36675644 10.3390/jcm12020716 PMC9864697

[107] Naseri A, Morsali S, Sabahi Z, Kakaei J, Hakimzadeh Z, Hamidi S, et al. The effects of Melatonin supplementation on cognitive outcomes in multiple sclerosis: a systematic review. Multiple Scler Relat Disorders. 2023;80: 105167. 10.1016/j.msard.2023.105167.

[108] Morsali S, Sabahi Z, Kakaei J, Hakimzadeh Z, Hamidi S, Gholipour-Khalili E, et al. Clinical efficacy and safety of melatonin supplementation in multiple sclerosis: a systematic review. Inflammopharmacology. 2023;31(5):2213–20. 10.1007/s10787-023-01271-4. 37429996 10.1007/s10787-023-01271-4

[109] Razmaray H, Nasiri E, Vakilipour P, Morsali S, Moradi A, Ebrahimian A, et al. The effects of melatonin supplementation on neurobehavioral outcomes and clinical severity in rodent models of multiple sclerosis; a systematic review and meta-analysis. Inflammopharmacology. 2024;32(2):927–44. 10.1007/s10787-023-01414-7. 38252220 10.1007/s10787-023-01414-7

[110] Bandehagh H, Gozalpour F, Mousavi A, Ghavshough MH. Effects of melatonin on the management of multiple sclerosis: a scoping review on animal studies. AIMS Med Sci. 2024;11:137–56. 10.3934/medsci.2024012.

[111] Bocheva G, Bakalov D, Iliev P, Tafradjiiska-Hadjiolova R. The vital role of melatonin and its metabolites in the neuroprotection and retardation of brain aging. Int J Mol Sci. 2024;25(10);10.3390/ijms25105122 10.3390/ijms25105122 PMC11121732 38791160

[112] Mogavero MP, Lanza G, Bruni O, DelRosso LM, Ferri R, Ferini-Strambi L. Sleep counts! Role and impact of sleep in the multimodal management of multiple sclerosis. J Neurol. 2023;270(7):3377–90. 10.1007/s00415-023-11655-9. 36905413 10.1007/s00415-023-11655-9

[113] Fleming WE, Pollak CP. Sleep disorders in multiple sclerosis. Semin Neurol. 2005;25(1):64–8. 10.1055/s-2005-867075. 10.1055/s-2005-867075 15798938

